# An Innovative Approach for Decision-Making on Designing Lifestyle Programs to Reduce Type 2 Diabetes on Dutch Population Level Using Dynamic Simulations

**DOI:** 10.3389/fpubh.2021.652694

**Published:** 2021-04-29

**Authors:** Teun Sluijs, Lotte Lokkers, Serdar Özsezen, Guido A. Veldhuis, Heleen M. Wortelboer

**Affiliations:** ^1^Department of Microbiology and Systems Biology, Netherlands Organisation for Applied Scientific Research (TNO), Zeist, Netherlands; ^2^Methodology Department, School of Management, Radboud University, Nijmegen, Netherlands; ^3^Department Military Operations, Netherlands Organisation for Applied Scientific Research (TNO), The Hague, Netherlands

**Keywords:** system dynamic model, type 2 diabetes, lifestyle, cost-benefit, patient journey modeling, decision support model

## Abstract

The number of individuals suffering from type 2 diabetes is dramatically increasing worldwide, resulting in an increasing burden on society and rising healthcare costs. With increasing evidence supporting lifestyle intervention programs to reduce type 2 diabetes, and the use of scenario simulations for policy support, there is an opportunity to improve population interventions based upon cost–benefit analysis of especially complex lifestyle intervention programs through dynamic simulations. In this article, we used the System Dynamics (SD) modeling methodology aiming to develop a simulation model for policy makers and health professionals to gain a clear understanding of the patient journey of type 2 diabetes mellitus and to assess the impact of lifestyle intervention programs on total cost for society associated with prevention and lifestyle treatment of pre-diabetes and type 2 diabetes in The Netherlands. System dynamics describes underlying structure in the form of causal relationships, stocks, flows, and delays to explore behavior and simulate scenarios, in order to prescribe intervention programs. The methodology has the opportunity to estimate and simulate the consequences of unforeseen interactions in order to prescribe intervention programs based on scenarios tested through “what-if” experiments. First, the extensive knowledge of diabetes, current available data on the type 2 diabetes population, lifestyle intervention programs, and associated cost in The Netherlands were captured in one simulation model. Next, the relationships between leverage points on the growth of type 2 diabetes population were based upon available data. Subsequently, the cost and benefits of future lifestyle intervention programs on reducing diabetes were simulated, identifying the need for an integrated adaptive design of lifestyle programs while collecting the appropriate data over time. The strengths and limitations of scenario simulations of complex lifestyle intervention programs to improve the (cost)effectiveness of these programs to reduce diabetes in a more sustainable way compared to usual care are discussed.

## Introduction

Diabetes mellitus (DM) is a complex disorder (1–3), and the number of individuals suffering from DM is dramatically increasing worldwide from over 360 million individuals in 2015, expected to be 500 million individuals in 2030 (3–5). Nine out of 10 individuals of the total DM population suffers from type 2 diabetes (T2DM) (6), which is characterized by a systemic metabolic disorder of which fasting blood glucose concentration slowly increases over years (1), and a major cause of slow healing foot ulcers, kidney failure, blindness, heart attacks, stroke, and lower limb amputation (3). T2DM can be treated and prevented, and as such, its consequences can be avoided or delayed by means of diet, physical activity, medication, as well as regular screening and treatment for complications (7). Without effective lifestyle intervention programs, DM results in an increasing burden on society and a substantial rise in healthcare costs (8, 9).

Also in The Netherlands, there is talk of an emerging epidemic, as over the years, the total population of DM patients increased from 3.9% in 2007 to 6.6% in 2019 (10, 11). Today, with a population of ~18.2 million people, 1.2 million people (6.6%) are estimated to suffer from diabetes, and it is estimated that this prevalence will increase ~30% by 2030 (12, 13). In latest calculations, the direct care costs of DM accumulated to a staggering 1.6 billion euros in 2017, accounting for around 1.8% of the total healthcare budget of The Netherlands (6, 14). There is an urgent need to explore how this rising societal burden can be reduced (cost)effectively. Recently, nutrition and lifestyle intervention programs focusing on treatment and especially reversing T2DM patients to a healthy state have been described in the literature (3, 15–17). Nevertheless, despite having access to intervention programs and knowledge of lifestyle on the onset and progression of T2DM and on total cost, only limited long-term policies on early prevention of pre-diabetes or T2DM patients are being implemented in the Dutch healthcare system. A clear understanding of the “T2DM patient journey,” our denomination of the journey from being healthy to becoming pre-diabetic and eventually becoming diabetic over time and possibly recovering from T2DM could support decision makers to optimize current policy including weigh-offs in social cost that is inferred through the accumulation of this (pre)diabetic population over time. An easily accessible simulation tool to bridge the gap between all these dimensions and to simulate interconnected effects of different programs on the outcome prior to the implementation of intervention strategies appears to be hardly existent in the literature.

Currently available simulation models for cost effectiveness or cost–benefit analysis of intervention programs on diabetes are often based upon Markov-type simulations of healthcare costs (18, 19). However, these Markov-type simulations do not offer an opportunity to estimate the consequences of unforeseen dynamic interactions over multiple years in the prescription of actions/interventions based on scenarios tested through “what-if” experiments (20). As such, dynamic simulation modeling could be a more effective method for designing especially high-leverage policy population lifestyle programs in the context of a complex problem such as type 2 diabetes. A small number of system dynamics (SD) models simulating the onset and progression of diabetes on population level over time have been described (21, 22). However, these SD models focus on the United States and therefore lack specific parameters for The Netherlands. The models were constructed for the purpose of explaining the progression of the number of patients with type 2 diabetes and did not take into account any form of dynamic simulation of the costs and benefits of lifestyle policies.

Therefore, in the present study, we aim to develop an SD model of the T2DM patient journey visualizing and exploring the consequences via “what-if” scenarios. To achieve this, we aim to simulate the costs and benefits of lifestyle programs over time via coupling three interconnected elements, i.e., (1) the dynamic journey from healthy (normoglycemic) to type 2 diabetes at population level, (2) the effect of lifestyle programs focusing on either prevention, treatment, and/or reversing T2DM, and (3) their associated total cost for society. The resulting simulation model does not have the aim to be 100% accurate in predicting the future, as this will not be possible. It aims to support the decision-making on T2DM pre- and intervention strategies by society and policy makers by means of a scenario-simulation tool of the cost and benefit analyses of type 2 diabetes lifestyle programs.

To develop a T2DM patient journey SD model, a thorough understanding of the core concepts of the onset and progression of the disease and associated costs of this complex social issue is crucial, which is described in section Background. The Methods section elaborates on the methods used to collect the appropriate data, model building, and addresses the role of feedback governing the system's behavior. The Result section provides an overview of the T2DM patient journey model, followed by the validation process of the model and the resulting scenarios. The Discussion section describes the limitations of the study and summarizes the most important insights resulting from the present study, followed by section Conclusion.

## Background

### The T2DM Patient Journey

T2DM is a complex disease, slowly evolving and both genetic and lifestyle related (1, 3). The individual is diagnosed with T2DM when fasting blood glucose or 2-h post-load blood glucose reaches values ≥ 7.0 and ≥11.1 mmol/L, respectively (23). The individual is referred to as “pre-diabetic” or “having pre-diabetes” when the fasting blood glucose or 2-h post-load blood glucose is higher than the blood glucose levels of healthy individuals but lower than the standard set to receive the diagnosis DM (2, 24, 25). Researchers have shown that T2DM and pre-diabetes results from an interaction of heritable risk factors and non-heritable risk factors (2, 5, 26–28). The non-heritable risk factors indicate the possibility of T2DM patients, and diagnosed pre-diabetes patients could be reversed to healthy individuals.

In line with this interaction, Loos and Janssens (29) argued that most individuals develop T2DM as a consequence of suffering from a metabolic syndrome or obesity, which predominantly occurs due the combination of an unhealthy lifestyle and a genetic susceptibility for gaining weight. In The Netherlands, the Heart Foundation and the Rotterdam R cohort study suggest that one out of three obese Dutch individuals will develop T2DM over time (30). The number of DM patients in The Netherlands has increased from 160,000 individuals in 1990 to 1.2 million in 2018 (12). In more recent years, Dutch healthcare is seemingly regaining control over the T2DM disease. Statistics Netherlands (31) has published data concerning the number of T2DM patients of the total Dutch population from 2014 to 2018 showing a steady change from 3.7% in 2014 to 4.0% in 2019. A faster rate of increase was observed in the two decades before (from 1990 to 2011), the number increased from 160,000 to 830,000 (10, 31), which is 1% to around 4.9% of the total population (31). A unified scientific explanation for this is not yet present; however, explanations might be found in more awareness of unhealthy lifestyle via media and success rates of the installment of local integrated lifestyle programs and bariatric surgery for especially obese T2DM patients (32). Nevertheless, the number of T2DM and pre-diabetes patients is foreseen to grow (25, 33), largely as a result of two factors. Besides the effect of increasing obesity (29, 33), an increasing aging population also results in an increasing T2DM population, as insulin resistance increase with obesity (34) and age (35). Currently, 51% of the Dutch population above 20 years old are overweight, of which 15% are obese. Both populations have increased over the past 5 years (31), and with the knowledge that one of three obese people develops T2DM, these numbers indicate that combined (cost)effective lifestyle programs may have extensive positive implications for society.

### Treatment of T2DM and Lifestyle Programs Aiming to Reverse T2DM

In The Netherlands, T2DM treatment is performed in compliance with the guidelines of the NHG-standard (de Nederlandse Huisartsen Genootschap Standaard in Dutch). Patients with T2DM are primarily treated in primary care by general practitioners (GPs) and their practice staff, including practice nurses (PNs). After medical treatment and lifestyle advice, every 3–6 months, routine diabetes consultations are performed, including physical examinations, blood glucose checks, and other laboratory tests, and the results discussed with patients. Both GPs and PNs perform medical and lifestyle counseling according to generally acknowledged criteria (36, 37). Besides treatment, researchers suggest that non-heritable risk factors are modifiable, and hence, assuming that individuals are able to make sustainable lifestyle changes, T2DM patients could be helped to permanently lower their blood glucose levels to normal levels. This process is referred to as “reversing the T2DM patient” (2). However, to achieve an actual reversal of T2DM patients, long-term behavioral changes are needed to restore normal, sustainable blood glucose levels (2, 3, 38–40). Reversal programs of T2DM are focused on providing treatment with limited use of insulin, as early routine use of insulin therapy can have negative consequences such as increased mortality, weight gain, increased risks of cancer, and hypoglycemia (41). In the beginning, T2DM intervention programs ranged from programs focusing on improving current treatment programs in The Netherlands, as approved by the NHG-standard, by re-evaluating the screening process resulting in the possibility to intervene earlier (10). As knowledge increased, other T2DM programs were developed focusing on changing lifestyle factors, such as personalized nutrition (40, 42), enhancing physical exercise (43), or after bariatric surgery for individuals with severe obesity (32, 44).

Treatment effectiveness, however, is highly dependent on the patient's intrinsic motivation to deliberately make lifestyle changes. Long-term coaching is often outside of the GP's role, with the development of several coaching tools as a result (45). Additionally, through current advancements of technology, the possibility of determining pre-diabetes gives the ability to reduce the causes earlier instead of fighting the consequences. Overweight and obesity for instance are very well-known to increase the incidence of T2DM (34). Early reversals are to reduce the potential long-term cost due to the development of the T2DM population. Hence, the present study argues that lifestyle programs should focus on the pre-diabetes population as well.

### The Construction and Calculation of Total Cost Arising From T2DM

The total cost associated with T2DM are costs resulting from individuals suffering from undiagnosed pre-diabetes, diagnosed pre-diabetes, and T2DM. These contributions in healthcare cost are abatable and/or even preventable, as research suggests that non-genetic obesity is relatively preventable in the assumption that individuals can make sustainable long-term behavior changes (46). There are multiple types of costs that arise from T2DM.

First, as The Netherlands is a welfare state, the public authorities provide unemployment benefits to the disabled (47). In case of a T2DM patient becoming disabled, the individual may claim for benefits according to the Work and Income Act (WIA). Within the invalidity scheme (the WIA scheme), a distinction is made between full, permanent disability (in Dutch “Inkomensvoorziening Volledig Arbeidsongeschikten,” IVA benefit) and partial disability (in Dutch “Werkhervatting Gedeeltelijk Arbeidsgeschikten,” WGA benefit).

Second, the employer develops cost arising due to labor productivity loss of the individual suffering from T2DM, which eventually may result in absenteeism of the T2DM patient (9). Additionally, the cost resulting from an obligatory salary payment for disabled employees is considered. When an employee is declared partially or fully disabled in The Netherlands, the employer is obligated to pay the salary of the employee up to 2 years as regulated in the Employee Insurance Agency (47).

Lastly, every person is insured under the Healthcare Insurance Act (ZVW) and the Long-Term Care Act (Wlz). Both medical as well as non-medical cost arise due to the need for primary care, hospitalization, and medical support. The present study, therefore, recognizes medical costs resulting from ambulatory care, hospitalization, physiotherapy, tools and devices use, general practitioner care, and medical treatment (the latter being separated for the elderly as insulin in an elderly body adjusts differently and therefore needs additional treatment). Furthermore, cost arising from comorbidities of T2DM (48–51) are taken into account. Additionally, costs arising from managing organizations concerned with T2DM by T2DM patients' foundation associations are included.

### Connecting the Elements

To explore the effectiveness of policy interventions, these three interconnected elements (the development of type 2 diabetes, lifestyle programs focused on reversing T2DM, and associated cost) need to be coupled to each other to provide a holistic view on the likely development of interventions on the T2DM stock and subsequent cost over time. The possibility of reversing type 2 diabetes and reducing its subsequent number of patients, the evidence of the effectiveness of lifestyle programs, and the subsequent cost for society should be treated as a combination of direct and indirect costs. Additionally, these pre- and intervention programs are financially heavy and require long-term commitment of (health) professionals and patients. To put all these aspects in a computer model, simulating the dynamics of the future trend of type 2 diabetics and associated cost could possibly guide the way for a more informed decision-making as to which programs decisionmakers can consider implementing in a certain context for optimal result.

## Methods

### Choosing SD for Computational Modeling

To model the T2DM patient journey in The Netherlands, an SD modeling approach was applied for the following reasons. In 2016, Alouki et al. reviewed that many of the public health interventions fall short of their larger goals, as these interventions usually are based upon linear projections of retrospect data (52). Current economic evaluation methods are often insufficient to simulate dynamics of complex phenomena involved and to visualize the outcome of “what if?” experiments. Environment, behavior, and population change over time, where risk factors, states of uncertainty, healthcare professions, and intervention resources are continuously interacting with one another. When the latter phenomena result in chronic problems, these can be defined as “wicked problems” (53, 54). To address wicked problems to an extent in which they might become manageable, this research argues the need of a holistic, integral approach, easily accessible for stakeholders, that captures non-linear behavior of the complex system over time. SD modeling is well-suited to address this complexity and an appropriate method for identifying important leverage points and examination of possible interventions in complex problems in advance of real-life implementation and subsequent evaluation, which would otherwise be too costly (21, 55). Therefore, with the ultimate aim to develop a simulation tool, SD was used to build a first version of an SD model to gain insights in the development of the T2DM patient journey in The Netherlands under the current policy, under lifestyle programs as several intervention policies, and under programs that combine lifestyle intervention policies.

### Basics of SD Methodology Explained

**Figure 1:**
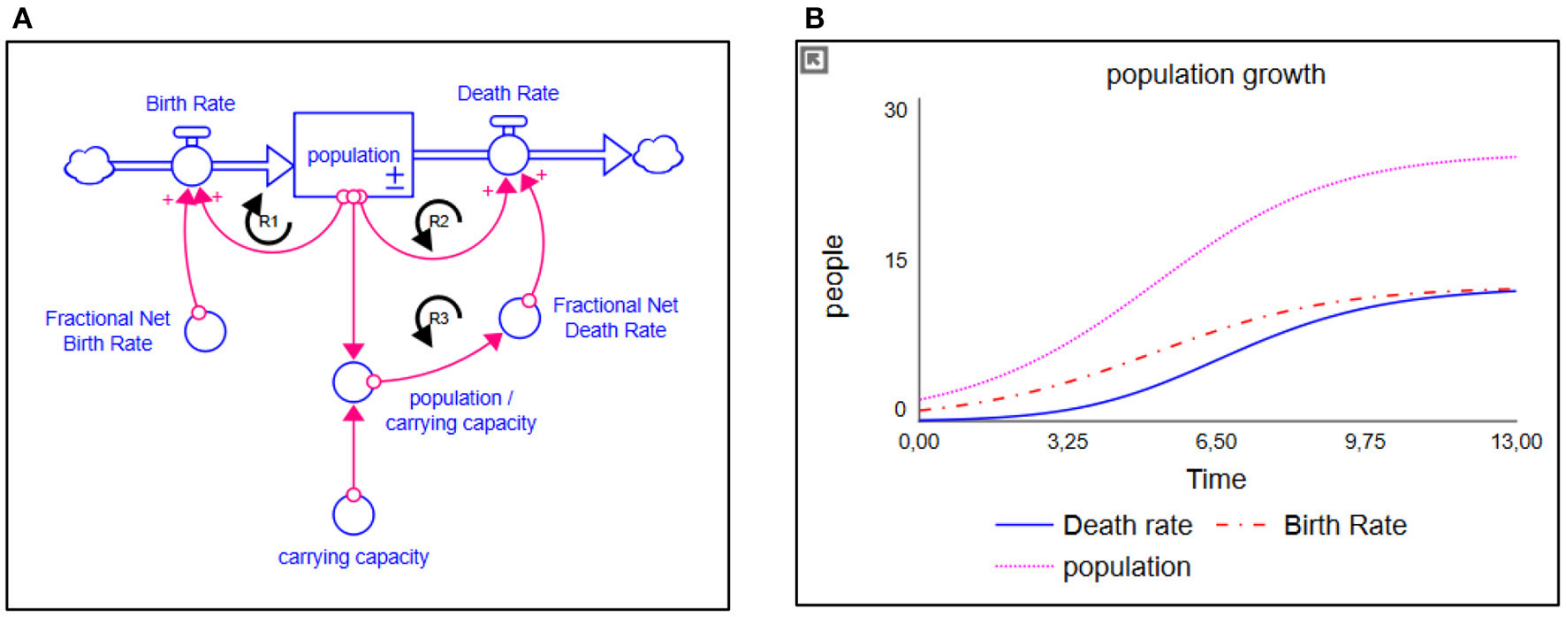
**(A)** Simplified population model and **(B)** its respective behavior. The stock is represented as a square; the flows are represented as double-lined arrows. The more people there are, the higher the birth rate is over time; a reinforcing loop (R1). Given a fixed carrying capacity, the more people there are, the more people will die; a counteracting reinforcing loop (R2). If the birth rate remains larger than the death rate, we will experience population growth. Population growth, however, has its limits due to all kinds of factor. Therefore, a balancing structure is built in, indicated by limiting variable “carrying capacity.” The closer the population gets to its respective carrying capacity, the more deaths will occur (a second counteracting reinforcing loop (R3), eventually stabilizing population growth where the amount of deaths equals the amount of births.

SD modeling simulates complex dynamic behavior over time and works with interrelationships, which are represented in multiple linked differential equations (56–59). The method predominantly makes use of stocks and flows. A simplified population model and its respective behavior, illustrating stocks and flows, are presented in Figure 1A. A stock, indicated with a square, represents a (accumulation of a) quantity at a certain point in time. A flow, however, indicated with a double-lined arrow, models the change in a stock over time (and therefore “flows” in or out of the stock). Variables, indicated by words preceding single arrows, add detail to the relations between stocks and flows. These variables mostly are endogenized within the system. Ultimately, the interaction and feedback (known as “loops”) between multiple endogenous variables, stocks, and flows create the dynamics over time (Figure 1B).

### Building the T2DM Patient Journey Model

**Figure 2:**
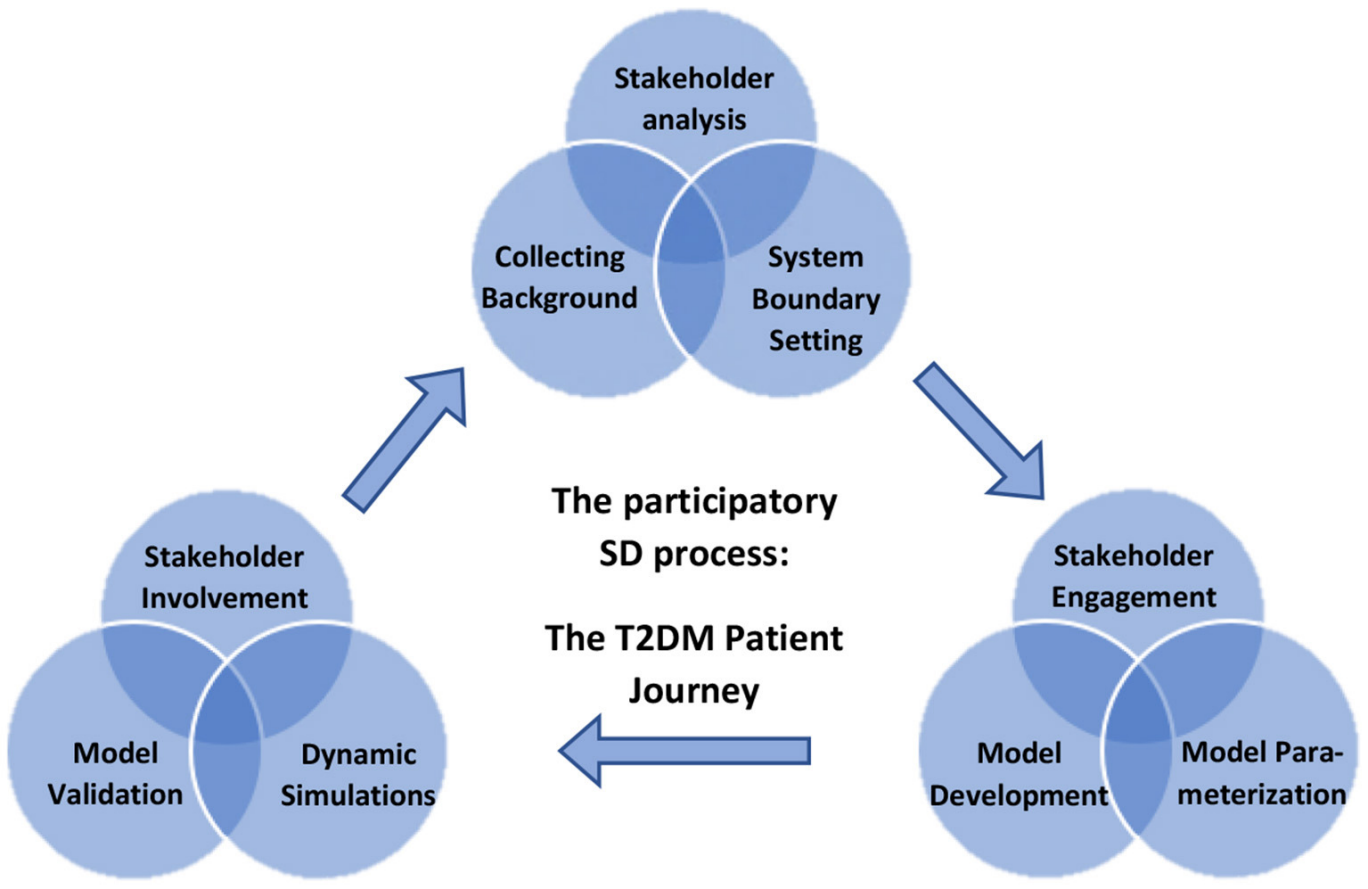
Overview of the participatory process of building a system dynamic (SD) model for decision-making on designing lifestyle programs to reduce type 2 diabetes on Dutch population level using dynamic simulations.

A participatory process was used to develop the SD model, as illustrated in Figure 2, and described below.

The current SD model finds its origins in the systemic analysis of diabetes performed by the Center for Disease Control and Prevention in the USA. The aim of this research by Homer et al. was to model the key drivers of population flows and inputs amenable to policy intervention (21). In a further adaptation of Homer's work, Jones et al. (22) redefined the model to a “patient journey.” A large focus point in the study of Jones et al. was the division of complicated and uncomplicated diabetes, and no attention was paid to associated costs. In the current study, we chose to focus on the impact of lifestyle intervention programs, which has the most impact on early prevention and reversal of diabetes, and as such, we did not distinguish between complicated T2DM and uncomplicated T2DM but focused on four additional factors affecting the early onset and actual emergence of T2DM. The factors were (1) the effect of an aging population (indicated in the model as Elderly fraction of adult population), (2) the effect of obesity on the emergence of T2DM (indicated in the model as Obese fraction of adult population), (3) the effect of population growth (indicated in the model as Adult growth rate), and (4) the T2DM death rate compared to death rate of the normoglycemic population (indicated in the model as Death rates for diabetic- and non-diabetic population, respectively). Multiple improvements were made due to new information available on the effect of prevention and lifestyle intervention programs changing the recovery rates of the subpopulations due to further evidence of reversing T2DM patients by nutrition and lifestyle. The structure of the T2DM model was parameterized toward the current situation and data available in The Netherlands.

### Data Collection to Build the Final T2DM Patient Journey Model

To collect the appropriate data, experts were iteratively interviewed to elicit their knowledge on T2DM and lifestyle intervention programs and to validate the relationships presented in the preliminary model, as described by Vennix (57). Accordingly, interviews were held with five scientific experts working at The Netherlands Organisation for Applied Scientific Research (TNO). All experts had detailed scientific knowledge and/or together more than 40-year experience on T2DM as well as its lifestyle programs in The Netherlands. Background of the experts were in biomedical sciences, health sciences, molecular biology, economics, and social history with a female/male ratio of 2/3. The expert interviews were conducted according to an interview guide approach, audio-recorded and analyzed for overall themes, as described by Vennix (57, 60). During the expert interviews, some changes in the preliminary model were suggested. For example, in contrast with Jones et al. (22), an outflow from the T2DM population to the normoglycemic population was suggested, as experts argued the possibility of reversing both pre-diabetics and T2DM patients. After crosschecking with the relevant literature review and background information as described the modules *The T2DM Patient Journey, Treatment of T2DM and Lifestyle Programs Aiming to Reverse T2DM*, and *The Construction and Calculation of Total Cost Arising From T2DM*, the preliminary model was modified accordingly. Thereafter, the modified preliminary SD model was validated by the experts. Finally, after several critical discussions, a final structure of the model was generated and inserted in Stella Architect software version 2.0.1 (iSee Systems, 2020). For parameterizing the model, data were extracted from scientific literature, World Bank, and Dutch databases of Statistics Netherlands (CBS), Dutch Healthcare Authority (NZa), The National Health Care Institute (ZIN), Netherlands Institute for Health Services Research (NIVEL), National Institute for Public Health and the Environment (RIVM), Dutch Diabetes Research Foundation (Diabetes Fonds), Dutch Diabetes Association (DVN), and the Netherlands Organization for Applied Scientific Research (TNO). Based on these data, the model was adjusted to represent the baseline scenario. Details of the data used are given in Supplementary Data.

## Results

### The Construction of the T2DM Patient Journey Model

**Figure 3:**
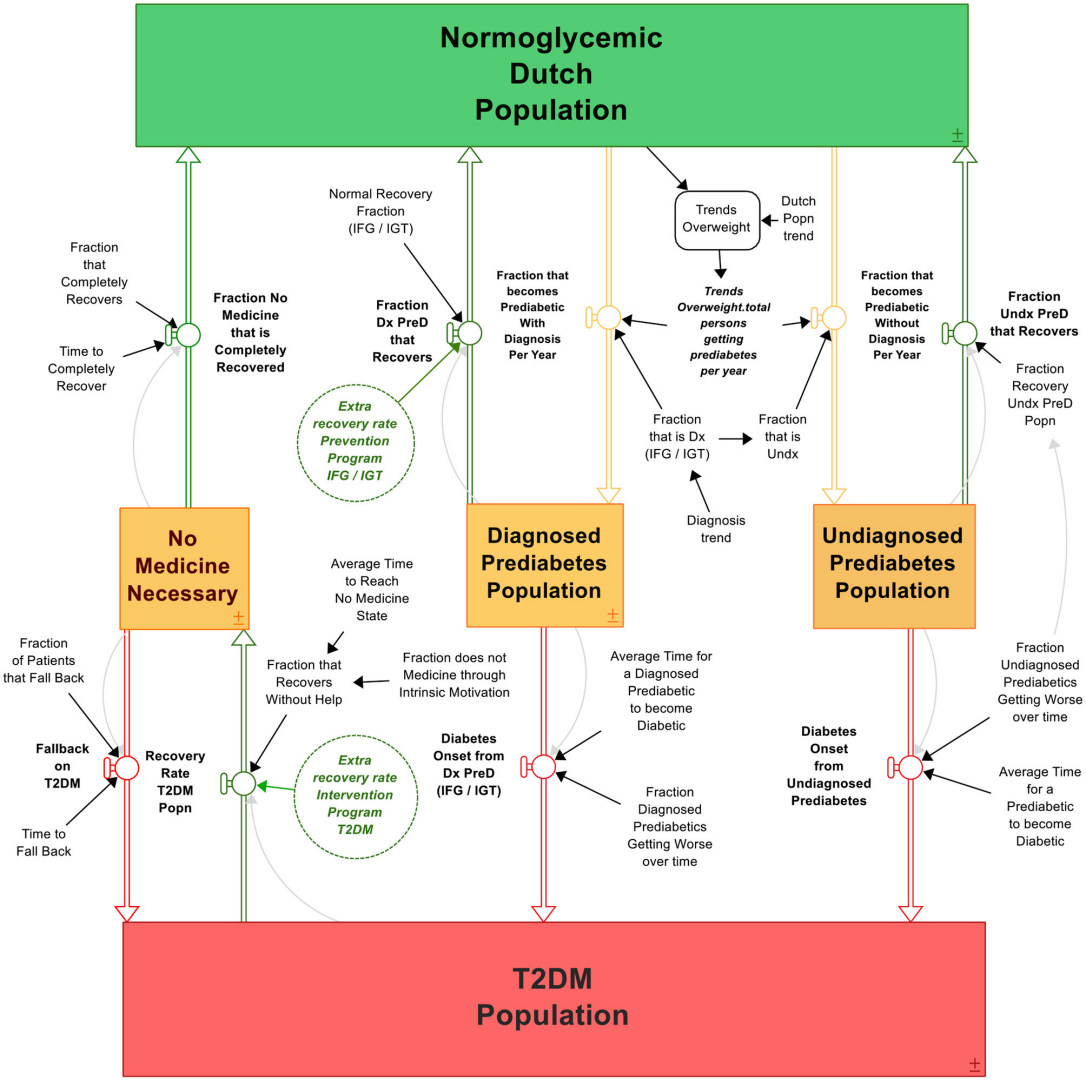
The T2DM patient journey. Boxes are stocks that accumulate over time, double arrows are in- or outflows that either give input or take output of the stock. Circles are variables that affect the in- or outflows, containing parameter values. Clouds are outputs that “flow out of the system” like deaths. The process is visualized from green (healthy) to orange (likely to develop T2DM) to red (developed T2DM), with multiple possibilities to bounce back in the system to “healthy” (green arrows). The system contains the differential equations as described in section The Construction of the T2DM Patient Journey Model.

The T2DM patient journey model displays the journey of individuals developing T2DM and the possibilities to reduce T2DM via interventions programs, coupled to the costs associated with T2DM and these subsequent programs in The Netherlands (Figure 3). The more type 2 diabetics, the higher the cost for society. Details of the model are given in Supplementary Data.

The model, as presented in Figure 3, is constructed via the following rationale. The initial stock is the “Normoglycemic population” of The Netherlands, consisting of all individuals able to develop T2DM. That is, the total Dutch population minus the T2DM population as T1DM patients are able to develop insulin resistance (61). Individuals are recruited to this population because of being born, migration, and passing away (formed as an exogeneous variable to keep the scope of focus on T2DM development: the variable “Dutch population trend”). A small fraction of this stock is already diabetic, whereas a small but growing fraction develops T2DM over time. Therefore, the initial values of the normoglycemic population plus type 2 diabetic population must equal the number of citizens in The Netherlands at the model's initial starting time. Under the circumstance when the Dutch population growth is equal to the growth projections of CBS, the model portrays the following behavior: the more diabetics, the larger the group of diabetics becomes, and the more the stock of normoglycemic population depletes; a reinforcing (+) relationship. On the other hand, the more type 2 diabetic people recover (or differently said, the stock “type 2 diabetics” reduces), the larger the normoglycemic group: a balancing (–) relationship. To reduce the amount of type 2 diabetics, one can “adjust” these flows with intervention strategies. We can reduce the amount of type 2 diabetics by either implementing a prevention program, which will reduce the amount of people becoming diabetic (slow down the reinforcing structure) or we can accelerate the type 2 diabetic recoveries by implementing intervention strategies (increase the balancing structure). From normoglycemic to diabetic, most individuals undergo generally the following process: first, one becomes overweight or even obese (outliers are represented as “non-obese”) and becomes a pre-diabetic. Either the person gets diagnosed or does not, represented as the “Diagnosed pre-diabetics” and “Undiagnosed pre-diabetics” stocks. Over time, a small fraction of both stocks “reverts” and flows back to the Normoglycemic population stock. Hence, individuals who suffered from either pre-diabetes or T2DM are again recruited to the normoglycemic population through “recovery.” However, a large part of these groups becomes type 2 diabetic, flowing into the “Type 2 diabetics” stock. A percentage of this type 2 diabetics population can be reversed, flowing back to the “Normoglycemic” stock. The according differential equations can be found in the Supplementary Data; the equations follow the rational:

(1) Diabetes population = (fraction of diagnosed pre-diabetics that become diabetic/time it takes to become diabetic for diagnosed pre-diabetics + fraction of undiagnosed pre-diabetics that become diabetic/time it takes to become diabetic for undiagnosed pre-diabetics).
(2) Normoglycemic population = [initial normoglycemic population + (fraction of type 2 diabetics that recovers/time it takes to recover)].

### Pre-diabetics

Individuals experiencing symptoms of pre-diabetes per definition first develop undiagnosed pre-diabetic and are thus recruited to the “Total persons that become pre-diabetics.” These pre-diabetes patients either can see a general practitioner and receive either the diagnosis impaired fasting glucose (IFG) or impaired glucose tolerance (IGT) (diagnosed pre-diabetes population, depicted in **Equation 2**), or leave the diagnosis to rest. The diagnosis rate (IFG and IGT) is determined by combining the fraction incidence (IFG) and the fraction incidence (IGT). Individuals suffering from either IFG or IGT are recruited from the total pre-diabetes population to the diagnosed pre-diabetes population when receiving the diagnosis IFG or IGT. Both individuals who are diagnosed and undiagnosed (or who are not visiting a general practitioner) can experience symptoms of T2DM, as the disease progresses over time. Fractions of both the diagnosed and undiagnosed pre-diabetes patients recover from pre-diabetes and is recruited to the normoglycemic population again; the latter has a smaller percentage of returning as people simply do not know they have pre-diabetes.

### Reversing the T2DM Population

Individuals are recruited over time to the T2DM population from the undiagnosed and diagnosed pre-diabetes population (IFG/IGT). T2DM patients leave the T2DM stock in two ways: first, passing away due to T2DM when reaching the average life expectancy of a T2DM patient; second, by recovering from T2DM. The fraction that recovers from T2DM determines the recovery rate of the T2DM population and thus the number of people recruited again to the normoglycemic population. Furthermore, a fraction of T2DM patients that are reversed to a state in which they do not need any medicine experience a relapse into the type 2 diabetes stock. This percentage is around 65% and heavily affects the follow-through of the success rates of a certain lifestyle program (48). With a bariatric surgery, however, this relapse percentage is only 5% (62). As CBS only provides data of T2DM from 2011 onwards, the initial value (*t* = 0) is defined by the Dutch population trend multiplied by the fraction of T2DM over the total population in 2011. Following the equations, the resulting SD model can be found in Figure 3.

### Dynamics Over Time

As SD modeling is a method to capture behavior over time, these differential equations are constructed accordingly by dividing the difference over a certain time step (dt) in this study in months. In the SD model, certain time factors are crucial for the behavior of the model, namely, the time a person takes on average to become pre-diabetic, the average onset and recovery times from (un)diagnosed pre-diabetics to either type 2 diabetic or normoglycemic population, and the recovery time for a type 2 diabetic to revert to a normoglycemic person. The time and fraction in which one transitions from one stock to another (in years) are represented in Table 1.

**Table 1:** Model values used that determine the time and fraction of individuals to transit from one stock to another.

**Transition**		**Average time** **(years)**	**Fraction of stock from A to B, divided by average time (DT)**
**From stock A**	**To stock B**		
Normoglycemic population (non-overweight)	Total persons that become pre-diabetics	2	0.01
Normoglycemic population (overweight)	Total persons that become pre-diabetics	3	0.33
Diagnosed pre-diabetics	Type 2 diabetics	4	0.70
Undiagnosed pre-diabetics	Type 2 diabetics	7	0.90
Diagnosed pre-diabetics	Normoglycemic population	2	0.30
Undiagnosed pre-diabetics	Normoglycemic population	4	0.10
Type 2 diabetics	Normoglycemic population	3	0.01
No Medicine necessary	Type 2 diabetics	2	0.65
No Medicine Necessary	Type 2 diabetics (Bariatric Surgery)	10	0.05

*Data are based upon assumptions made from the literature, reports, and discussion with experts. Fractions of stocks are indicated between 0 and 1, in which 1 indicates 100%. Data are based upon assumptions made from the literature, reports and discussion with experts*.

### Submodules

In order to perform (cost)effectiveness analyses with the SD model, two types of submodules were developed and connected to the dynamics of the T2DM population: the submodule on effectiveness of the lifestyle intervention programs on reducing the pre-diabetic and T2DM population and a submodule on the costs and benefits of the lifestyle intervention programs. The submodules run simultaneously of each other and complement themselves with elements of the “larger” T2DM model. The T2DM model forms merely an input for the cost for society (the more patients, the more the cost, and the more program cost, the more total cost). The effectiveness of the lifestyle programs, however, enjoys its input through the T2DM population (for example, the higher the T2DM population, the more people to treat) and, in turn, provides a feedback for the T2DM population dynamics (the more effective the lifestyle program, the more people recover from T2DM).

### Submodule 1: Intervention and Prevention Programs

The structure enabling the implementation of intervention programs is presented in the Supplementary Data. This module serves as an input for a possible higher recovery rate of T2DM patients (prevention programs) as well as a lower onset rate from the non-glycemic to pre-diabetic stock. The rationale is as follows. Assuming that individuals are able to make sustainable lifestyle changes, the present study argues that T2DM patients (either IFG or IGT) can either recover without intervention program (normal recovery rate) and with the support of one (recovery rate intervention program). The recovery rate intervention program is determined by the program recruitment rate and the program success rate. The number of individuals recruited to participate in a certain intervention program is determined by the potential recruitment rate or the indicated recruitment rate, as it is initialized with the special-function MIN. The potential recruitment rate can only be determined by the maximum patients able to receive treatment over time. Furthermore, the indicated recruitment rate represents the actual number of T2DM patients who are recruited to participate in the certain program, which is determined by investments. Thereafter, the model with implementation of a certain lifestyle intervention program is connected to the associated costs and benefits.

### Submodule 2: Total Cost of Type 2 Diabetes

**Figure 4:**
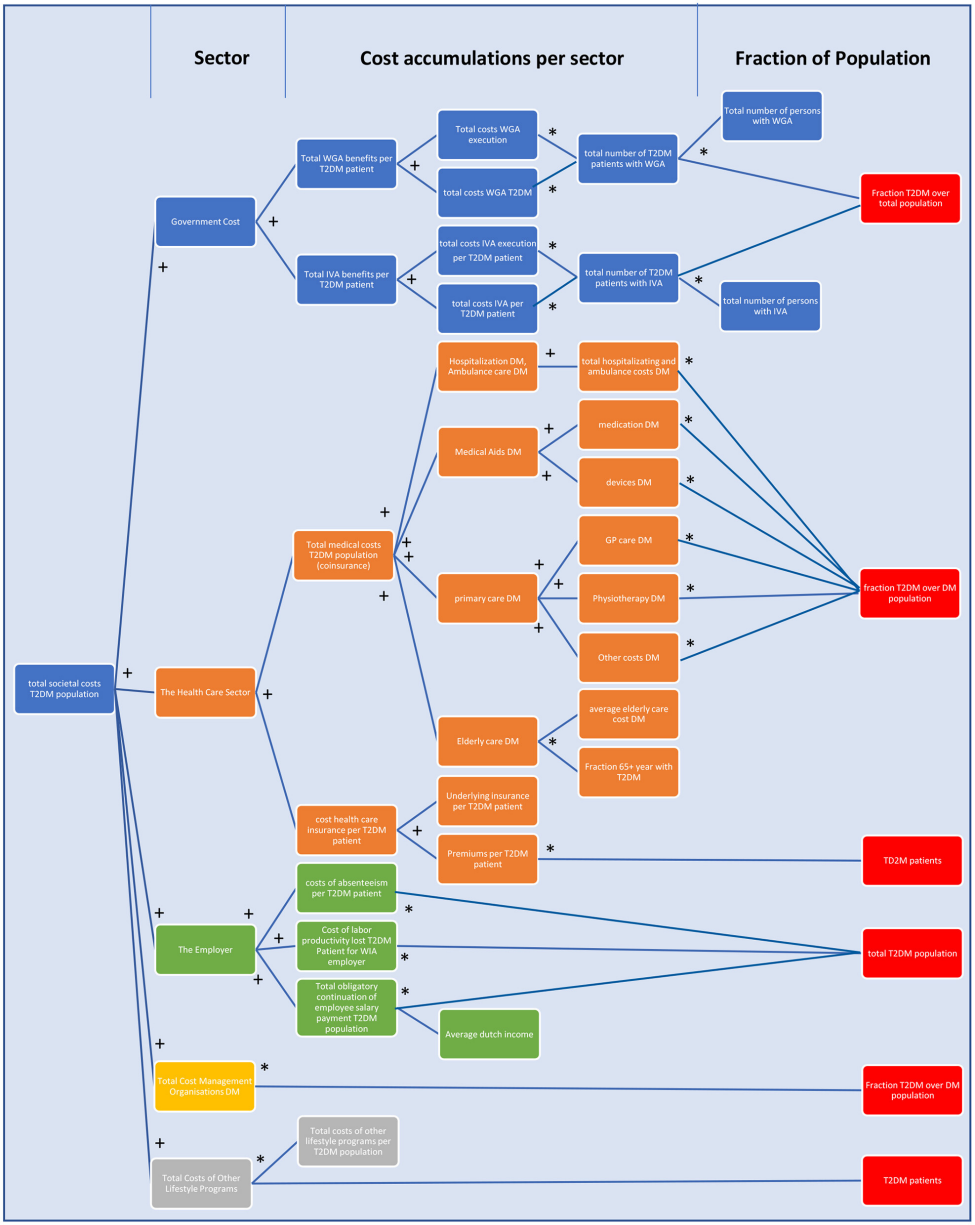
A schematic overview of the total costs of T2DM in The Netherlands as incorporated in the model. The figure follows the logic: from the portion of the Dutch population that has T2DM, in whichever way that fits for the equation (red), multiple costs to society are calculated via multiplications (*) and cumulations (+). Blue = costs for Dutch authorities, orange = costs for the financial health care sector, green = costs for the employer, yellow = total costs for management of DM, and gray = total costs of other lifestyle programs. For details of WGA, WIA, and IVA, see section The Construction and Calculation of Total Cost Arising From T2DM (47).

The total cost of type 2 diabetes in The Netherlands consist of costs for the Dutch authorities, the health insurer, the employer, and, more implicitly to a certain extent, the patient. In Figure 4, a schematic and static overview is given of the costs, as they have been incorporated in the model and their respective mathematical relationships. The total accumulative costs are represented in the Supplementary Data. In order to couple the costs to the development and scenario setting of T2DM and lifestyle programs over time, it is required to couple elements as the T2DM development over time (the type 2 diabetics stock) to these static equations.

### Model Validation

**Figure 5:**
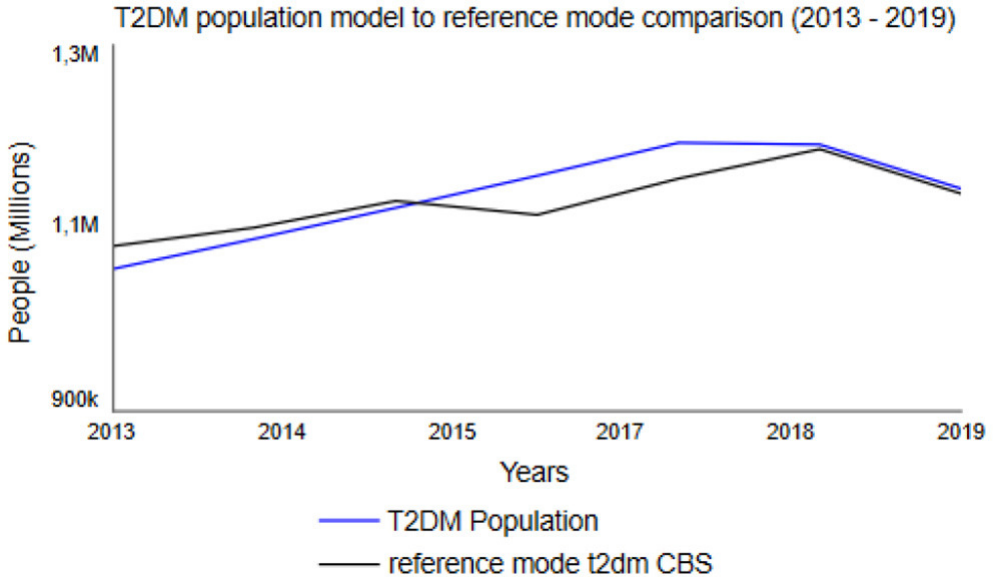
Dutch T2DM Population between 2013 and 2019, based upon Statistics Netherlands (CBS) data (31) as reference mode (black line) and simulated change using the T2DM patient journey model (blue line).

In SD modeling, model validation is a combination of a social, qualitative process as well as formal testing (63). Therefore, it is argued that model validity should be judged considering the model's purpose (63). Accordingly, the T2DM patient journey model's validity was judged considering its purpose by conducting several structure tests and structure-behavior-oriented tests as described earlier (26, 64). Within structural behavior testing, an extreme condition test was used to examine whether the model still rationally portrays the right behavior when being put to extreme measures. In the model, the normoglycemic population was set to both 0 and 100 million, whereas the model adequately portrayed reality in for example cost: 0 T2DM patients resulted in 0 costs, 100 million T2DM patients resulted in a few billion euros in costs. By conducting behavior sensitivity analysis, all exogenous variables were examined based on their impact on the model's behavior (59). When conducting behavior sensitivity analysis on *Fraction Individuals Developing Pre-diabetes* (see Supplementary Data), for example, the simulations showed that all five populations are very sensitive to changes in the fraction. The model appears to be very sensitive for changes regarding the fraction of individuals determining the number of overweight and obese individuals who develop diabetes [*Fraction Overweight Developing Pre-diabetes (BMI)*]. Furthermore, the behavior reproduction test examined the model's ability to replicate the reference mode of behavior (59) on CBS published data on the normoglycemic population and the T2DM population from 2013 to 2019 (31). When comparing the model's behavior and the CBS data, roughly the same behavior was observed (Figure 5). Hence, the model is able to replicate the reference mode of behavior.

### Scenario Simulation of the Cost and Benefits of Future Lifestyle Programs

The T2DM patient journey model is simulated for a 45-year time period from 1990 to 2035 to enable comparison with results presented in the relevant literature (10). Following the current policy, simulation shows that the total costs of T2DM increase as a result of an increase in the type 2 diabetics stock, as shown by the relationship between the T2DM population and the total costs for society T2DM population. As all the elements are interconnected and validated to “sufficiently correct with reality,” policy levers are implemented in the model to examine dynamic cost–benefit interactions in the model. The current policy levers are five-fold: First, one can choose to “activate” a certain program, either prevention or an intervention (pre-diabetes program and T2DM program). Second, one can determine the success rate of the program. Realistically, this is usually a trade off with cost: the more it is effective, the more people are needed so the more cost, which is another policy lever (cost multiplier). The potential amount of people that will be focused on can also be determined with “potential patients to recruit in program.” A program can choose to either focus on a small group and make this extremely efficient or draw it to a wider group with the risk of being less efficient.

**Figure 6:**
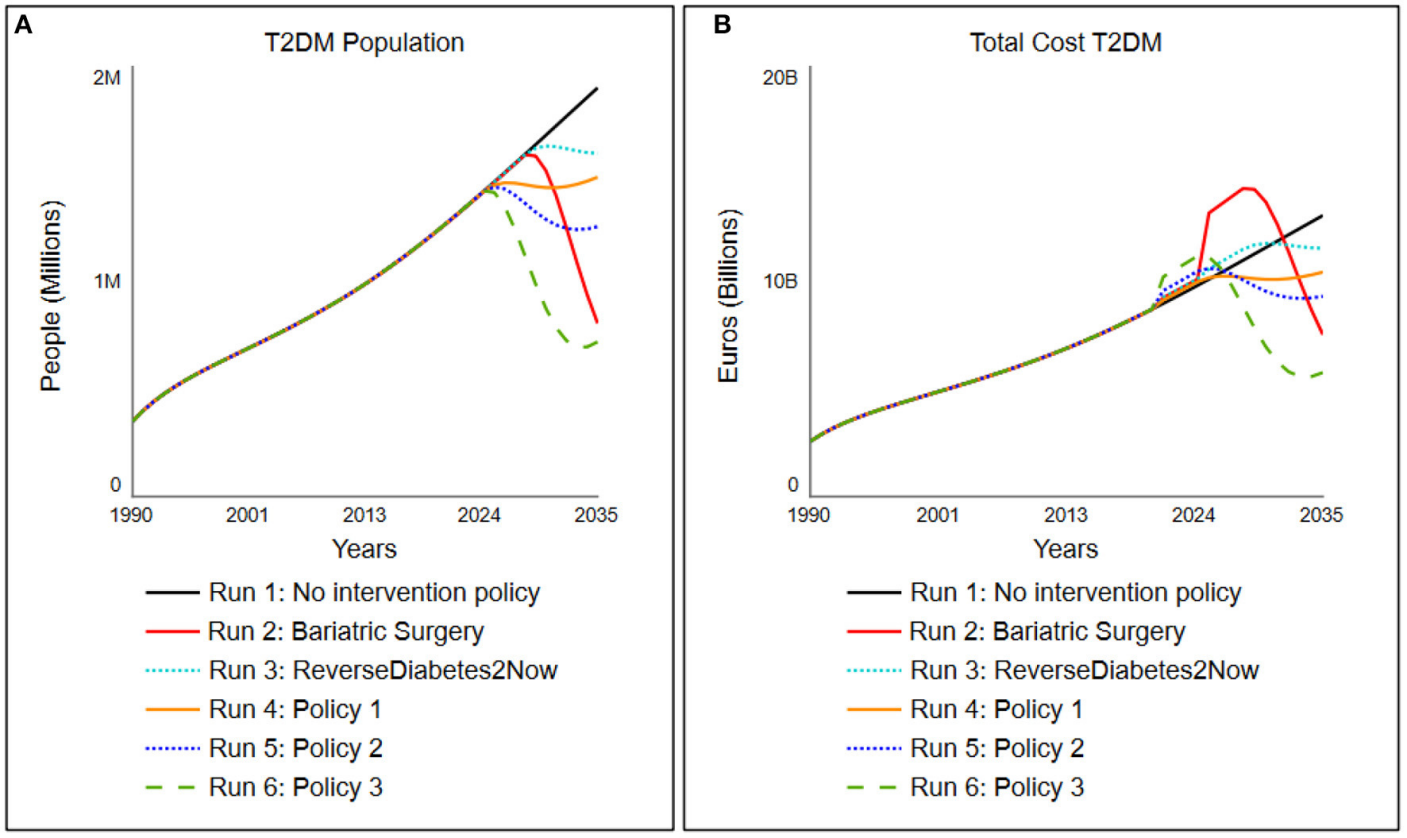
Simulated change in **(A)** the total cost for society associated with T2DM population and **(B)** the total T2DM under five different scenarios tested, which are activated in 2020 (details of scenarios indicated in Table 2).

Using different “what-if?” experiments, the development of the T2DM population and the total costs for society of T2DM were simulated under hypothesized multiple policy scenario runs: current policy (“No lifestyle program”) and policy options 1–4 (activated in the year 2020), as to be seen in Table 2, followed by the outcomes of these particular policy parameterizations to total cost for society of all the interventions (Figure 6A) and its subsequent T2DM population (Figure 6B).

**Table 2:** Hypothesized intervention scenarios and their parameter values used in scenario simulation.

**Simulated intervention scenarios**	**T2DM program**				**Pre-diabetes program**			
	**Yes/no**	**Fraction** **recruited**	**Success** **rate**	**Cost** **multiplier**	**Yes/no**	**Fraction** **recruited**	**Success** **rate**	**Cost** **multiplier**
No Intervention policy	No	–	–	–	No	–	–	–
Policy 1 “Bariatric Surgery”	Yes	0.28	0.95	17	No	–	–	
Policy 2 “ReverseDiabetes2Now”	Yes	0.002	0.28	1	No	–	–	–
Policy 3	Yes	0.3	0.3	1.0	No	–	–	–
Policy 4	Yes	0.45	0.3	2.0	Yes	0.3	0.3	1.0
Policy 5	Yes	0.6	0.5	3.0	Yes	0.4	0.5	3.0

*Parameter values are indicated between 0 and 1, in which 1 indicates 100%. Data are based upon assumptions made from literature, reports and discussion with experts*.

### Scenario Run 1: No Intervention Policy

In the “no intervention policy” situation, we simulate that no additional lifestyle program is yet installed. Therefore, the program activations remain 0. The model shows that the inflows diabetes onset rate from undiagnosed and pre-diabetes, diabetes onset rate from diagnosed pre-diabetes (IFG/IGT) continuously exceed the outflows recovery rate T2DM population and recovery rate (un)diagnosed pre-diabetes, resulting in a steadily increasing T2DM population. The total cost for society associated with T2DM follow the trend of the T2DM population and result in current setting to a cost for society in the Netherlands that increases to 13 billion euros in 2035.

### Additional Intervention Policies

When an additional intervention program is implemented, it will always incur an extra cost. However, T2DM patients can also fully recover via a lifestyle change without the support of any additional lifestyle programs (current policy), although rare. To determine (cost)effectiveness of additional lifestyle programs as offset under increased cost, several policy scenarios (different lifestyle programs) are run on the framework of the T2DM patient journey and its subsequent societal costs. Multiple aspects are being taken into account in all these additional lifestyle interventions: the success rate of a program, the choice of a focused (T2DM population only) or an integrated program (both T2DM and pre-diabetes population), the potential amount of patients (either T2DM only or T2DM and pre-diabetes) that can be recruited in the program, and the cost per person of the program. All lifestyle programs contain trade-offs. For example, to have a lifestyle program with success rate while still “keeping it cheap,” one needs a focused approach with a selective group as personalized care is expensive. Another example is the trade-off of having an integrated approach that covers most of the society; in order to not make the lifestyle program too expensive, one needs to make trade-offs in the success and recruitment rates of the program.

#### Scenario Run 2: Bariatric Surgery

Scenario run 2 is one where a bariatric surgery is performed on patients. The success rates are based on Yska et al. (44), and the cost for this surgery is based on RIVM data (62). In this policy, an adaptation on the relapse rate and time from the “no medicine necessary” stock is made. The parameter values for this group are set on relapse rate of 5% (0.05 as compared to 0.65) with a delay of 10 years instead of 2. The cost of this bariatric surgery, however, accumulates to around 17 times the amount of a lifestyle intervention, ranging from 15,000 to 20,000 euros (62). The success rate of this policy is set on 95%. Since only people with BMI above 35 are eligible for bariatric surgery due to Dutch care regulations, the percentage of people recruited is set to 28%, which roughly accounts for the percentage of people who have a BMI above 35. The policy portrays that, however extremely effective, this is a very expensive policy option when it is deployed over the larger population (Figure 6, run 2).

#### Scenario Run 3: The “ReverseDiabetes2Now” program

In scenario run 3, the program of “ReverseDiabetes2now” is simulated with its current success rates (40) and gradually increased in population treatment over 10 years. The program currently has a success rate of 28% where no medicine is necessary, and around 2,500 people are treated yet (roughly 0.002% of T2DM population in 2020). Pricing is assumed to be set on “average.” The model simulates, when the treated population is gradually increased over 10 years to 30% of the entire T2DM population, that the type 2 diabetes population will stagnate and subsequently its cost (Figure 6, run 3).

#### Scenario Run 4: Policy Option 1

Policy option 1 is a moderately successful additional lifestyle program (success rate of the program is 30%, or 0.3) but merely focuses on the T2DM population. To keep the cost down (average cost), the program can only focus on 30% of the T2DM population. As a result, the program turns out to level off the costs in the year 2025 (for a closer look, simulation of the cost for society of the policy run is presented in Figure 6, run 4). Although it practically levels off the growth of the T2DM population, the program is still a high societal burden in terms of cost.

#### Scenario Run 5: Policy Option 2

Policy option 2 is a fairly successful additional integrated program (success rate of the program is 45% or 0.45 for both T2DM patients as well as 30% or 0.3 for pre-diabetes billion by 2) for T2DM patients and standard (1.0) for pre-diabetes patients. As a result, the program brings down the T2DM population to a far extent and is a relatively small investment for the benefit it brings (Figure 6, run 5: a small bump in 2020, which is being corrected through an extensively lower T2DM population and thus a decrease in total cost for society of around two billion euros in 7 years). The combination turns out to be successful for total cost for society in the long run while ensuring that only a small investment needs to be made for the society to implement this integrated program.

#### Scenario Run 6: Policy Option 3

Policy option 3 is an expensive (pricing multiplied by 4) yet very successful additional program focused on the T2DM population (success rate of the program is 60% or 0.6 for T2DM patients). As mentioned, personalized care is expensive, hence four times the standard costs. One can see a drastic decrease in the T2DM population. In terms of long-term gain, this is the most successful option for both reduction in T2DM population as total cost, but more practically, this comes at a price. The stakeholders need to preinvest as much as 1 billion euros in this program (Figure 6, run 6: a large bump in 2020 of 1 billion euros compared to current policy) to get this result, which likely will result in large resistance of stakeholders.

## Discussion

This study was performed to demonstrate how dynamic simulations can be used to explore the consequences of policies in order to design lifestyle intervention programs of a complex disease like T2DM based on scenarios tested through “what-if” experiments. At first, understanding of the patient journey from a normoglycemic and/or obese health state toward pre-diabetes toward type 2 diabetes, and vice versa, was based upon literature data, databases, and expert interviews. Thereafter, a detailed study to capture available cost for society, current policy, and available T2DM intervention programs were collected. The combination of literature and empirical data, discussions with knowledge experts, and several publicly accessible data sources in The Netherlands, appeared to be successful for developing a first version of an SD-based simulation tool focusing on the patient journey of type 2 diabetes as a complex reversal disease, and the associated total cost for the society. The analyses presented in this study demonstrate the insights and conclusions one could draw from building an SD model and use it for simulation experiments. The experiments improved understanding of some characteristic dynamics of the simulated diabetes population in The Netherlands: (1) obesity is a major factor driving the growth of pre-diabetes and as such T2DM prevalence; (2) the inability of the current policy to reduce T2DM prevalence in the long term; (3) lifestyle intervention programs focusing on T2DM alone reduce T2DM population on short term but is less effective over longer time; and (4) the significant delays between primary prevention efforts and downstream improvements in diabetes outcomes. These results indicate the need for additional intervention programs focusing on pre-diabetics.

The participatory process of building simulation models can help improve our understanding of a complex chronic disease dynamics like type 2 diabetes. In addition, it can also identify the missing data needed to improve the model and our knowledge and may enhance the commitment of collaborative participatory action research to collect longitudinal data for evaluation of the intervention programs. As a consequence, this may result in more cost-effective lifestyle intervention designs. However, all simulation models have several inherent limitations. All models remain incomplete simplifications of the reality, and their conclusions are affected both by structural boundaries and by the uncertainties of the data with which they are calibrated (59). Techniques such as boundary critique and sensitivity testing (59, 64) can be used to assess the extent to which models may be affected by these simplifications and uncertainties. In the case of the type 2 diabetes patient model, sensitivity testing suggests that the magnitudes of its simulated futures, such as those seen in Figure 1, are subject to some imprecision because of uncertainties about input parameters but that the directions of change and thus our general findings are unaffected by these uncertainties.

### Limitations

To calibrate the model, accessible databases in The Netherlands of, among others, CBS, KPMG, Nederlandse Zorgautoriteit, NIVEL, RIVM, and TNO were used. For some concepts, data estimates and assumptions in trends and rate had to be made due to limited data availability or conflicting datasets. No distinction in age groups was made, as specific data for all other variables per age group were scarce. Age is considered to have a limited effect, as the fraction of individuals below the age of 20 years old suffering from T2DM is close to 0 (31). The average life expectancy of a healthy individual in The Netherlands is 81.7 years (31). Assuming that undiagnosed individuals unaware of their T2DM condition pass away sooner than individuals aware of their condition, a shortened lifespan of 10 and 8 years was considered for the undiagnosed pre-diabetes population and the diagnosed pre-diabetes population, respectively. For the T2DM population, a shortened lifespan of 9 years was assumed.

Publicly accessible data on sustainable recovery rates are not available yet. It is assumed that already recovered individuals and normal healthy individuals develop T2DM at the same pace. For both diagnosed pre-diabetes populations, as the disease has not yet progressed as much, it is assumed that the motivation to recover of diagnosed pre-diabetes patient suffering from IFG or IGT is present, and thus, the recovery fraction for the IFG/IGT diagnosed pre-diabetes population was set at 10%. Furthermore, it was assumed that undiagnosed pre-diabetes patients are unaware of their condition and thus are the least motivated to recover or simply do not know about their disease until it is too late. Therefore, the recovery for undiagnosed pre-diabetes population was set at 5%.

In the submodule total cost for society, some costs were determined for the total DM population in accordance with the relevant literature, the costs for the T2DM population were determined by taking the product of the total cost for society of the total DM population and the fraction that 9 out of 10 DM patients suffer from T2DM (6, 31). Additionally, it was assumed that all T2DM patients and all pre-diabetes patients are employed.

### Suggestions for Future Work

For the full potential of the T2DM patient journey model, it is clear that extra longitudinal data need to be collected to improve the prognostic value for decision-making on designing lifestyle programs to reduce type 2 diabetes on Dutch population level. However, based upon the available data, the model showed that compared with the current situation, the policy options 3 and 4 are observed to be the most effective in terms of decrease in T2DM population and reduction in total cost for society. This is due to these policy options having the highest reference frame—these programs apply to the entire diabetes patient journey. Therefore, we believe that future research should focus on identifying the most optimal policy options under a similar approach. That is, conducting scenario analysis with individual policy success rates per group in the patient journey in a certain context taking constrictions into account, while collecting the data as indicated in Table 2 over time to evaluate the lifestyle program while improving the T2DM patient journey model.

The present study argues that both creation of awareness on pre-diabetes, combined lifestyle programs, and collective actions are important to reduce T2DM, as besides the current expenditure of billions of euros that are largely avoidable, it could contribute to positive societal change and reverse obesogenic tendencies in Dutch society. Policy should not only focus on medication or lifestyle intervention programs in the case of severe symptoms but also focus on early prevention programs by supporting sustainable change toward a healthy diet, exercise, sleep pattern, and reduction in chronic stress, for a full remission toward a healthy state. Currently, individuals deliberately need to make lifestyle changes to manage their blood glucose levels, whereas a more supportive role from society and public authorities to reduce unhealthy stressors would be beneficial. Over the years, some changes have been made, such as promoting daily exercise and installing bike lanes. At the same time, however, grocery stores sell unhealthy products at eye level, and too often, stairs are installed behind doors while elevators are prominently placed at the main entrance of office buildings. Dutch citizens are therefore receiving contradictory prompts from their social environment, and a tension appears to exist between incentives that promote healthy and unhealthy lifestyles.

To parameterize the model to reality, assumptions had to be made. In accordance with (65), each assumption is explicitly stated in the Supplementary Data and open for criticism. Therefore, future studies should be focused on discussion of these assumptions by gaining knowledge and longitudinal data on the development of (un)diagnosed pre-diabetes and the recovery process of (un)diagnosed pre-diabetes patients. This will also result in less parameter sensitivity in the behavior sensitivity analysis.

Additionally, when assessing the effectiveness of the policy options, the underlying assumption is that that all individuals are able to make sustainable lifestyle changes that continue beyond the timeframe of the model. However, future studies should focus on including the effect of this assumption for two reasons. First, the majority of T2DM patients are elderly (35). Consequently, those individuals have practiced unhealthy lifestyles for a long period of time. Hence, it could be argued that this limits the success of adopting a new lifestyle. Second, as T2DM is more progressed than (un)diagnosed pre-diabetes (1), it is arguable that it is harder to make behavioral lifestyle changes for T2DM patients than for (un)diagnosed pre-diabetes patients. Another assumption that should be challenged in future research is to measure the effect of stakeholders' budget to investment in lifestyle programs.

Moreover, future studies should include the effect of gender, education level, social status, migration background, and culture, as research suggests the factors to be of significant influence in the development of T2DM (66, 67). With more accurate data of the local situation, the model has the potential to support local policy makers with new insights and hypotheses *ex ante* implementation of prevention and lifestyle intervention programs for more sustainable (cost)effectiveness in their own region. However, even with their inevitable imprecision and incompleteness, simulation models can enhance learning and decision-making, which is their primary purpose (59).

## Conclusion

In the present study, we used SD modeling to build a prototype model to be implemented into a simulation tool to gain a clear understanding of the type 2 diabetes mellitus patient journey and to assess the impact of lifestyle intervention programs on total cost for society associated with developing, preventing, and lifestyle treatment of pre-diabetes and diabetes. We were able to capture the extensive knowledge of diabetes, current available data on both population level, lifestyle intervention programs, and associated cost for society in The Netherlands into one simulation model. The model was able to visualize and measure the relationship between leverage points on the different outcomes of lifestyle programs. When assessing the policy options' effectiveness in terms of their total cost for society reductions, the model shows that simulation runs 5 and 6 prove to be most effective in terms of long-term societal cost reduction and simulation run 1 is the least effective one. As the systemic approach shows, this is due to focus points in the model: Simulation runs 2–4 are merely focused on the reduction in current T2DM cases, which is largely increasing cost as personalized care is expensive. Simulation runs 5 and 6 take the entire client journey into account and addresses a personalized option to both pre-diabetics and T2DM. In this way, more individuals with diversified problems get treated, preventing the number of individuals developing T2DM to a large extent and thus, with more focused effort, lower cost for society. However, a crucial remark on practicality should be made in the assessment. In simulation runs 2 and 6, although more effective for the T2DM population as well as cost in the long run, large sums of money are required to initiate and maintain the programs. Simulation run 5, however, although lower in success, requires a far lower initial investment and is still very effective in terms of reduction in the T2DM population. Simulation run 5 (or Policy option 4) is therefore, in a practical sense, considered the most effective policy option that is presented in this study.

To our knowledge, this is the first integrated simulation model of the type 2 diabetes patient journey in The Netherlands from the normoglycemic to the diabetes population. We believe that through scenario simulations of complex lifestyle intervention programs in an early stage of design and implementation process, the (cost)effectiveness of lifestyle intervention programs for diabetes scan be improved.

## Data Availability Statement

The datasets presented in this study can be found in online repositories. The names of the repository/repositories and accession number(s) can be found below: World Bank,Dutch databases of Statistics Netherlands (CBS), Dutch Healthcare Authority (NZa), The National Health Care Institute (ZIN), Netherlands Institute for Health Services Research (NIVEL), National Institute for Public Health and the Environment (RIVM), Dutch Diabetes Research Foundation (Diabetes Fonds), Dutch Diabetes Association (DVN) and the Netherlands Organization for Applied Scientific Research (TNO).

## Author Contributions

TS and LL: framework and model. HW, LL, and TS: literature review. TS, GV, and LL: methodology. SÖ: input. HW: supervision. TS: results, graphs, analysis, and conclusion. TS and HW: discussion and final edits. HW, GV, and SÖ: review. All authors contributed to the article and approved the submitted version.

## Conflict of Interest

The authors declare that the research was conducted in the absence of any commercial or financial relationships that could be construed as a potential conflict of interest.
