# Supplementary material for: An Innovative Approach for Decision-Making on Designing Lifestyle Programs to Reduce Type 2 Diabetes on Dutch Population Level Using Dynamic Simulations
Source: Front Public Health. 2021 Apr 29;9:652694. doi: 10.3389/fpubh.2021.652694 (PMC8116515; doi:10.3389/fpubh.2021.652694)
Supplement: Supplementary file 1 [file Data_Sheet_1.docx]

**Supplementary Data**

**The T2DM patient journey model**

| Run Specs | |
| --- | --- |
| Start Time | 1980 |
| Stop Time | 2040 |
| Time step (DT) | 1/1 |
| Fractional DT | True |
| Save Interval | 1 |
| Sim Duration | 1.2 |
| Time Units | Years |
| Pause Interval | 0 |
| Integration Method | Euler |

| Total | Count | Including Array Elements |
| --- | --- | --- |
| Variables | 149 | 149 |
| Modules | 1 |  |
| Sectors | 9 |  |
| Stocks | 5 | 5 |
| Flows | 9 | 9 |
| Converters | 135 | 135 |
| Constants | 66 | 66 |
| Equations | 78 | 78 |
| Graphicals | 11 | 11 |
| Macro Variables | 40 |  |

|  |  | *Equation* | *Properties* | *Units* | *Documentation* |
| --- | --- | --- | --- | --- | --- |
|  | **T2DM Patient Journey – Stocks** | | | | |
| 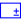 | Diagnosed_Prediabetes_Population(t) | Diagnosed_Prediabetes_Population(t - dt) + (Fraction_that_becomes_Prediabetic_With_Diagnosis_Per_Year - "Diabetes_Onset_from_Dx_PreD_(IFG_/_IGT)" - Fraction_Diagnosed_Prediabetics_that_Recovers) * dt | INIT Diagnosed_Prediabetes_Population = 40000 | Persons | The total diagnosed prediabetes population suffering from IFG/IGT (De Vegt et al., 2001, p. 2111, OptimaleGezondheid.com, 2020). Initial value in 1980 is not measured at that time – however calibrated to |
| 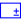 | No_Medicine_Necessary(t) | No_Medicine_Necessary(t - dt) + (Recovery_Rate_T2DM_Popn - Fraction_No_Medicine_that_is_Completely_Recovered - Fallback_on_T2DM) * dt | INIT No_Medicine_Necessary = 0 | Persons | In conversation with the expert S. Wopereis (2020), people firstly go through a stage where no medicine is necessary. Around 65% reverts back to the T2DM population during this stage. |
| 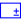 | Normoglycemic_Dutch_Population(t) | Normoglycemic_Dutch_Population(t - dt) + (Recovery_Rate_Undx_PreD_Popn + Fraction_Diagnosed_Prediabetics_that_Recovers + Fraction_No_Medicine_that_is_Completely_Recovered - Fraction_that_becomes_Prediabetic_Without_Diagnosis_Per_Year - Fraction_that_becomes_Prediabetic_With_Diagnosis_Per_Year) * dt | INIT Normoglycemic_Dutch_Population = Dutch_population_trend | Persons | Total population with normal glucose levels  (CBS, 2020). |
| 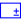 | T2DM_Population(t) | T2DM_Population(t - dt) + (Diabetes_Onset_from_Undiagnosed_Prediabetes + "Diabetes_Onset_from_Dx_PreD_(IFG_/_IGT)" + Fallback_on_T2DM - Recovery_Rate_T2DM_Popn) * dt | INIT T2DM_Population = 120000 | Persons | The total T2DM population is the total Dutch population times the fraction of T2DM population over total population  (CBS, 2020). |
| 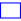 | Undiagnosed_Prediabetes_Population(t) | Undiagnosed_Prediabetes_Population(t - dt) + (Fraction_that_becomes_Prediabetic_Without_Diagnosis_Per_Year - Recovery_Rate_Undx_PreD_Popn - Diabetes_Onset_from_Undiagnosed_Prediabetes) * dt | INIT Undiagnosed_Prediabetes_Population = 40000 | Persons | Undiagnosed diabetes population is not measured. Initial is derived from the CBS data (2020) in combination with Baan et al (2009). |
| **T2DM patient journey - flows** | | | | | |
| 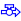 | "Diabetes_Onset_from_Dx_PreD_(IFG_/_IGT)" | (Diagnosed_Prediabetes_Population*Fraction_Diagnosed_Prediabetics_Getting_Worse_over_time)/Average_Time_for_a_Diagnosed_Prediabetic_to_become_Diabetic | - | Persons/Year | The rate at which individuals suffering from diagnosed prediabetes (IFG) develop T2DM per year. Data from the 2Diabeat study (2020). |
| 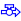 | Diabetes_Onset_from_Undiagnosed_  Prediabetes | Undiagnosed_Prediabetes_Population*Fraction_Undiagnosed_Prediabetics_Getting_Worse_over_time/Average_Time_for_a_Prediabetic_to_become_Diabetic | - | Persons/Year | The rate at which individuals suffering from undiagnosed prediabetes develop T2DM per year. Data from 2Diabeat study (2020) |
| 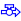 | Relapse_on_T2DM | (No_Medicine_Necessary*Fraction_of_Patients_that_Fall_Back)/Time_to_Fall_Back | - | Persons/Year | The rate of people that relapse from the No Medicine Necessary stock to the T2DM stock per year. |
| 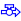 | Fraction_Diagnosed_  Prediabetics_that_  Recovers | ("Normal_Recovery_Fraction_(IFG_/_IGT)"*Diagnosed_Prediabetes_Population)+("Normal_Recovery_Fraction_(IFG_/_IGT)"*"Extra_recovery_rate_Prevention_Program_IFG_/_IGT") | - | Persons/Year | The rate at which individuals suffering from diagnosed prediabetes (IFG) recover from diagnosed prediabetes (IFG) per year. |
| 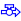 | Fraction_No_Medicine_that_is_Completely_Recovered | (No_Medicine_Necessary*Fraction_that_Completely_Recovers)/Time_to_Completely_Recover | - | Persons/Year | The rate of people that is completely recovered after an intervention program and flow back to the normoglycemic population. |
| 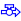 | Fraction_that_becomes_Prediabetic_With_Diagnosis_Per_Year | (Trends_Overweight.total_persons_getting_prediabetes_per_year)*"Fraction_that_is_Diagnosed_(IFG_/_IGT)" | - | Persons/Year | The fraction of people that flow from the normoglycemic stock to the diagnosed prediabetic stock. |
| 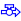 | Fraction_that_becomes_Prediabetic_Without_Diagnosis_Per_Year | Trends_Overweight.total_persons_getting_prediabetes_per_year*Fraction_that_is_Undiagnosed | - | Persons/Year | Number of individuals developing symptoms of prediabetes per year. |
| 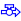 | Recovery_Rate_T2DM_Popn | (T2DM_Population*Fraction_that_Recovers_Without_Help)+(Extra_recovery_rate_Intervention_Program_T2DM) | - | Persons/Year | The rate at which individuals suffering from T2DM recover from T2DM per year. |
| 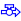 | Recovery_Rate_Undx_PreD_Popn | Undiagnosed_Prediabetes_Population*Fraction_Recovery_Undx_PreD_Popn | - | Persons/Year | The rate at which individuals suffering from undiagnosed prediabetes recover from undiagnosed prediabetes per year. |
| 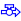 | Bariatric_flow | potential_T2DM_people_to_recruit_program*((No_Medicine_Necessary*fraction_to_fall_back_bariatric)/time_fall_back_bariatric) | - | Persons/year | The rate of individuals with a bariatric surgery that relapse from the no medicine necessary stock to the type 2 diabetes stock |
| **T2DM patient journey – variables** | | | | | |
| 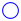 | Average_Time_for_a_  Diagnosed_Prediabetic_to_become_Diabetic | 3 | - | Years | According to Tabak et al (2009), it can take 5 to 10 years for an individual suffering from prediabetes to develop T2DM. It is assumed that individuals aware of their condition, that is individuals suffering from diagnosed prediabetes (IFG), develop T2DM slower than individuals unaware of their condition, that is. undiagnosed prediabetes patients. While looking at the model's behavior, it is assumed to be 3 years. It is assumed that it is similar for IFG and IGT individuals. |
| 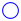 | Average_Time_for_a_  Prediabetic_to_become_Diabetic | 7 | - | Years | According to Tabak et al (2009), it can take 5 to 10 years for an individual suffering from prediabetes to develop T2DM. It is assumed that individuals aware of their condition, i.e. individuals suffering from diagnosed prediabetes, develop T2DM slower than individuals unaware of their condition, that is undiagnosed prediabetes patients. While looking at the model's behavior, it is assumed to be 7 years. |
| 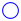 | Average_Time_to_  Reach_No_Medicine_  State | 3 | - | Years | In conversation with the expert S. Wopereis, the average time to reach the ‘no medicine’ state would be around 3 years. |
| 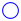 | Diagnosis_trend | GRAPH(TIME) | 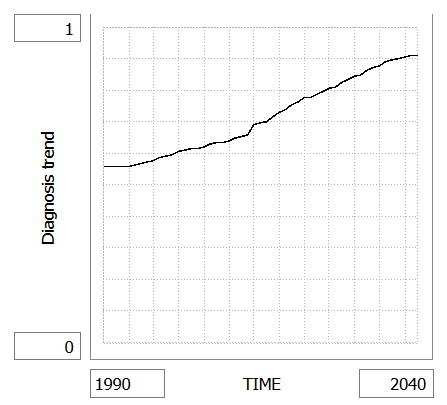 | Dimensionless | The amount of diagnosed people and projections thereof  (2Diabeat study, 2020) |
| 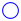 | Dutch_population_  trend | GRAPH(TIME) | 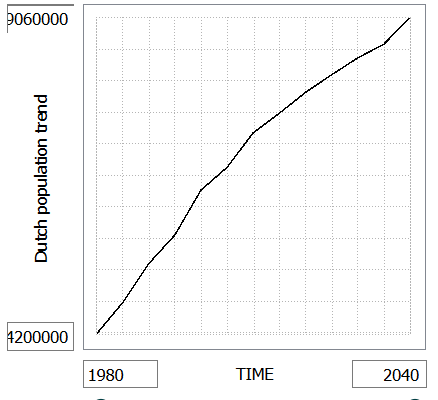 | Persons | prognosis CBS population growth in the Netherlands 2040:  19060000 (https://www.cbs.nl/nl-nl/nieuws/2019/51/prognose-19-miljoen-inwoners-in-2039)  population growth 1980-2020:  14 mil – 17.4 mil |
| 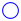 | Dx_PreD_Popn_2013 | 550000 | - | Persons | The number of people with diagnosed prediabetes in The Netherlands in 2013 (OptimaleGezondheid.com, 2014). |
| 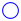 | "Extra_Recovery_Rate_Intervention_program_(IFG_/_IGT)" | "program_Success_Rate_(IFG)"*"Potential_Recruitment_Rate_(IFG)" | - | Persons/Years | The rate at which diagnosed prediabetes patients (IFG) are recovering with the support of the prevention program Lifestyle as Medicine (LaM) per year. |
| 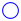 | "Extra_Recovery_Rate_Intervention_Program_(T2DM)" | "Potential_Recruitment_Rate_(T2DM)"*"program_Success_Rate_(T2DM)" | - | Persons/Years | The rate at which T2DM patients are recovering with the support of the intervention program LaM per year. |
| 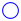 | Extra_recovery_rate_Intervention_Program_T2DM | ("Extra_Recovery_Rate_Intervention_Program_(T2DM)") | - | Dimensionless | The recovery rate that proceeds from the intervention program submodule. |
| 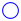 | "Extra_recovery_rate_Prevention_Program_IFG_/_IGT" | SMTH3("Extra_Recovery_Rate_Intervention_program_(IFG_/_IGT)"; 10) | - | Dimensionless | The program recovery rate is smoothed out over 10 years |
| 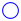 | Fraction_Diagnosed_Prediabetics_Getting_Worse_over_time | 0.70 | - | Dimensionless | According to statistics, around 70% of diagnosed prediabetics gets type 2 diabetes (2Diabeat study, 2020) |
| 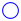 | Fraction_Dx_PreD_Popn_over_T2DM_Popn | Diagnosed_Prediabetes_Population/T2DM_Population | - | Dimensionless | Fraction diagnosed prediabetes (IFG) patients of the total T2DM population. |
| 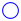 | "fraction_IFG/_IGT_2013" | 0.668 | - | Dimensionless | The fraction of diagnosed prediabetes patients in 2013  (RIVM, 2013) |
| 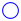 | Fraction_of_Patients_that_Relapse | 0.65 | - | Dimensionless | In conversation with the expert S. Wopereis, on average 60-70% falls back to the total T2DM population |
| 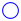 | Fraction_of_Patients_that_Relapse_Bariatric | 0.05 | - | Dimensionless | On average 5% falls back to the total T2DM population that have undergone a bariatric surgery. Based on Suijkerbuijk et al. (2013). |
| 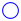 | Fraction_Recovery_Undx_PreD_Popn | 1-Fraction_Undiagnosed_Prediabetics_Getting_Worse_over_time | - | 1/Years | Fraction of individuals suffering from undiagnosed prediabetes that recovers per year.  It is assumed to be 0.05. |
| 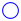 | Fraction_T2DM_over_DM_Popn | 0.9 | - | Dimensionless | It is assumed that 9 out of 10 individuals suffering from DM suffer from T2DM (Suiker in perspectief, 2013). |
| 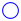 | Fraction_T2DM_over_Total_Popn | 0.035 | - | Dimensionless | The percentage T2DM patients in The Netherlands (CBS, 2017b). |
| 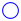 | Fraction_T2DM_Popn_over_Total_Popn | T2DM_Population/Total_Population_1 | - | Dimensionless | The fraction of T2DM patients of total population in The Netherlands. |
| 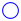 | Fraction_T2DM_Popn_over_Total_Popn_1 | T2DM_Population/Total_Population_1 | - | Dimensionless | The fraction of T2DM patients of total population in The Netherlands. |
| 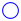 | Fraction_that_Completely_Recovers | 0.10 | - | Persons | Percentage of people that completely recovers from T2DM |
| 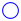 | "Fraction_that_does_not_Need_Medicine_through_Intrinsic_Motivation_/_medicine" | 0.001 | - | Persons/Years | The rate at which individuals suffering from T2DM recover without the use of LaM program per year. |
| 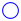 | "Fraction_that_is_Diagnosed_(IFG_/_IGT)" | Diagnosis_trend | - | 1/Years | Fraction undiagnosed prediabetes patients diagnosed with IFG per year (De Vegt et al., 2001, p. 2111). Fraction undiagnosed prediabetes patients diagnosed with IGT per year (De Vegt et al., 2001, p. 2111).  Trend over the years will increase, point 2001 is set on 0.668 whereas 2040 is set around 0.80. |
| 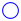 | Fraction_that_is_Undiagnosed | 1-"Fraction_that_is_Diagnosed_(IFG_/_IGT)" | - | Dimensionless | The fraction of people that is diagnosed with prediabetes |
| 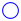 | Fraction_that_Recovers_Without_Help | SMTH3("Fraction_that_does_not_Need_Medicine_through_Intrinsic_Motivation_/_medicine"; Average_Time_to_Reach_No_Medicine_State) | - | Dimensionless | The fraction of people that recovers on intrinsic motivation |
| 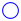 | Fraction_Undiagnosed_Prediabetics_Getting_Worse_over_time | 0.95 | - | Dimensionless | The fraction of undiagnosed prediabetics that get T2DM, estimated 5% recovers on the basis of expert conversation, leaving 95% to become worse. |
| 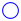 | "Normal_Recovery_Fraction_(IFG_/_IGT)" | 0.10 | - | 1/Years | Fraction of individuals suffering from diagnosed prediabetes (Impaired Fasting Glucose) that recovers per year. It is assumed to be 0.10. it is also assumed that the fraction of individuals recovering is similar for IFG and IGT individuals. |
| 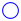 | Time_to_Completely_Recover | 2 | - | Years | Time it takes on average to completely recover from the ‘no medicine’ state to the Normoglycemic state, in accordance with S. Wopereis. |
| 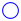 | Time_to_Relapse | 2 | - | Years | Time it takes on average to fall back from the ‘No medicine necessary’ state to the T2DM state, in accordance with S. Wopereis. |
| 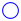 | Time_to_Relapse_bariatric | 10 | - | Years | Time it takes on average to fall back from the ‘No medicine necessary’ state to the T2DM state on a patient that has undergone bariatric surgery. Based on Suijkerbuijk et al. (2013). |
| 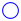 | "Treatment_Time_(T2DM)" | 1 | - | Years | The time it takes to treat one T2DM patient to become fully recovered.  Voeding Leeft lifestyle intervention program has a program that takes 6 months of intensive support and 18 months of after support (KeerDiabetesOm, 2017). |
| **Module - trends overweight** | | | | | |
| 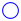 | heaviness_of_trend_reversal_1 | 1 | - | Dimensionless | **Policy lever –** The possibility to adjust the heaviness of a possible trend reversal in the future in obesity. Ranges from 0 – xx where 1 is the standard setting. |
| 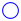 | heaviness_of_trend_reversal_2 | 1 | - | Dimensionless | **Policy lever -** The possibility to adjust the heaviness of a possible trend reversal in the future in ‘heavy overweight’. Ranges from 0 – xx where 1 is the standard setting. |
| 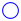 | heaviness_of_trend_reversal_3 | 1 | - | Dimensionless | **Policy lever -** The possibility to adjust the heaviness of a possible trend reversal in the future in ‘overweight’. Ranges from 0 – xx where 1 is the standard setting. |
| 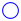 | heavy_overweight_persons_getting_prediabetes_per_year | (.Normoglycemic_Dutch_Population*("trend_conversion_to_yearly_growth_-_heavy_overweight"*"onset_rate_from_heavy_overweight_(BMI_30-35)")) | - | Persons/years | The amount of persons that get prediabetes per year (data from 2Diabeat study, 2020) |
| 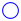 | heavy_overweight_trend | GRAPH(TIME) | 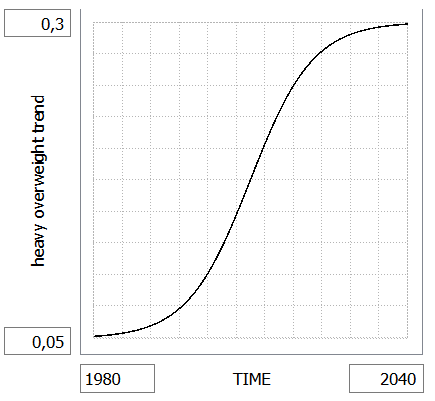 | Dimensionless | The trendline of heavy overweight in line with data from CBS on heavy overweight (CBS, 2020) and further projections from the 2Diabeat study (2020). |
| 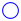 | "non-overweight_persons_getting_prediabetes" | "Non-overweight_population"*"Onset_Rate_from_Non-Obesity_1" | - | Persons/Years | The amount of persons that have no form of obesity, yet do get prediabetes. |
| 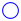 | "Non-overweight_population" | .Normoglycemic_Dutch_Population-(.Normoglycemic_Dutch_Population*"trend_conversion_to_yearly_growth_-obesity")-(.Normoglycemic_Dutch_Population*"trend_conversion_to_yearly_growth_-_heavy_overweight")-(.Normoglycemic_Dutch_Population*"trend_conversion_to_yearly_growth_-_overweight") | - | Persons | Population of individuals without obesity. |
| 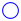 | obese_persons_getting_prediabetes_per_year_1 | (.Normoglycemic_Dutch_Population*("trend_conversion_to_yearly_growth_-obesity"*"Onset_Rate_from_Obesity_(BMI>35)")) | - | Persons/years | The amount of obese persons that get prediabetes. Data form the 2Diabeat study (2020). |
| 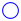 | obesity_trend | GRAPH(TIME) | 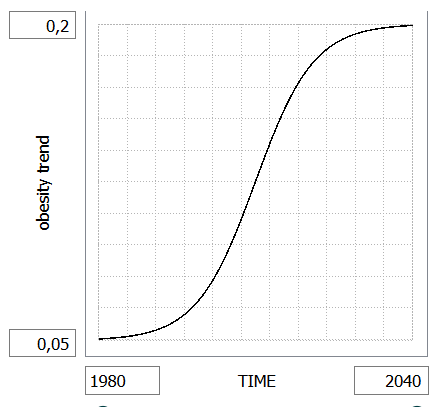 | Dimensionless | The trendline of obesity in line with data from CBS on obesity (CBS, 2020) and further projections from the 2Diabeat study (2020). |
| 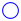 | "onset_rate_from_heavy_overweight_(BMI_30-35)" | 0.23 | - | Dimensionless | The percentage of persons that get prediabetes from heavy overweight. Data from the 2Diabeat study (2020). |
| 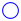 | "Onset_Rate_from_Non-Obesity_1" | 0.001 | - | Persons/Years | The percentage of persons that get prediabetes without having overweight. Data from the 2Diabeat study (2020). |
| 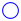 | "Onset_Rate_from_Obesity_(BMI>35)" | 0.33 | - | Persons/Years | The rate at which obese individuals are developing prediabetes per year. Data from the 2Diabeat study (2020). |
| 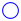 | "onset_rate_from_overweight_(BMI_25-30)" | 0.15 | - | Persons/Years | The percentage of persons that get prediabetes from heavy overweight. Data from the 2Diabeat study (2020). |
| 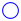 | overweight_persons_getting_prediabetes_per_year_1 | (.Normoglycemic_Dutch_Population*("trend_conversion_to_yearly_growth_-_overweight"  *"onset_rate_from_overweight_(BMI_25-30)")) | - |  | The amount of persons that get prediabetes from overweight). |
| 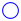 | overweight_trend | GRAPH(time) | 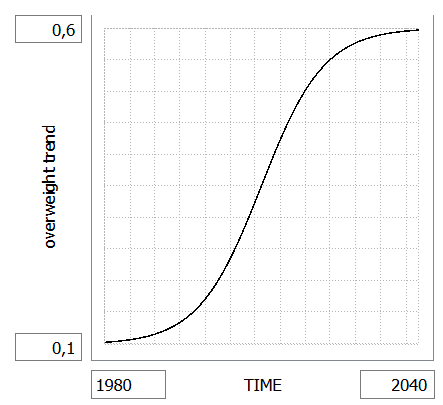 |  | The trendline of overweight in line with data from CBS on overweight (CBS, 2020) and further projections from the 2Diabeat study (2020). |
| 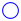 | simulation_time | 60 | - | Years | The simulation time in years, defining the trendline of obesity, heavy overweight and overweight. |
| 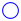 | total_persons_getting_prediabetes_per_year | (obese_persons_getting_prediabetes_per_year_1) + heavy_overweight_persons_getting_prediabetes_per_year +overweight_persons_getting_prediabetes_per_year_1 + "non-overweight_persons_getting_prediabetes" | - | Persons/Years | The total amount of persons getting prediabetes per year |
| 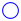 | "trend_conversion_to_yearly_growth_-_heavy_overweight" | IF "trend_reversal_on_-_heavy_overweight"= 1 THEN (IF TIME >2020 THEN (heavy_overweight_trend/simulation time)*trend_reversal_heavy_overweight ELSE heavy_overweight_trend/simulation_time) ELSE (heavy_overweight_trend/simulation_time) | - | Dimensionless | The conversion button on trend conversion of heavy overweight. When trend conversion is on, it simulates heavy overweight with the trend conversion. if trend conversion is off, the model proceeds with the regular behavior of heavy overweight. |
| 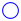 | "trend_conversion_to_yearly_growth_-_overweight" | IF "trend_reversal_on_-_overweight"= 1  THEN (overweight_trend/simulation_time)*trend_reversal_overweight  ELSE (overweight_trend/simulation_time) | - | Dimensionless | The conversion button on trend conversion of overweight. When trend conversion is on, it simulates overweight with the trend conversion. if trend conversion is off, the model proceeds with the regular behavior of overweight. |
| 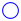 | "trend_conversion_to_yearly_growth_-obesity" | IF "trend_reversal_on_-_obesity"= 1  THEN (obesity_trend/simulation_time)  *trend_reversal_obesity ELSE (obesity_trend/simulation_time) | - | Dimensionless | The conversion button on trend conversion of obesity. When trend conversion is on, it simulates obesity with the trend conversion. if trend conversion is off, the model proceeds with the regular behavior of obesity. |
| 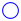 | trend_reversal_heavy_overweight | GRAPH(SMTH3(heaviness_of_trend_reversal_1; 10; 2020)) | 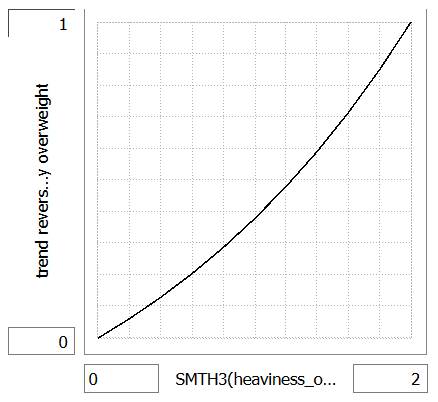 | Dimensionless | As the future is uncertain, the possibility to reverse the trend ‘heavy overweight’, also to be adjusted by the policy lever ‘heaviness of trend reversal’. |
| 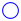 | trend_reversal_obesity | GRAPH(SMTH3(heaviness_of_trend_reversal_2; 10; 2020)) | 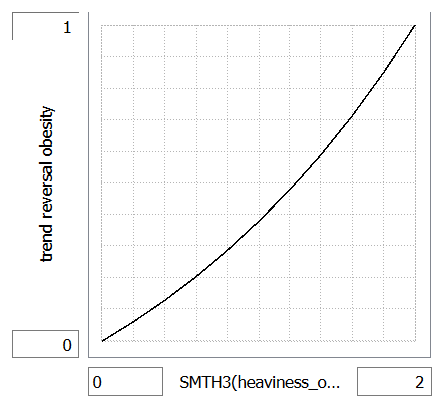 | Dimensionless | As the future is uncertain, the possibility to reverse the trend ‘obesity’, also to be adjusted by the policy lever ‘heaviness of trend reversal’. |
| 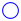 | trend_reversal_overweight | GRAPH(SMTH3(heaviness_of_trend_reversal_3; 10; 2020)) | 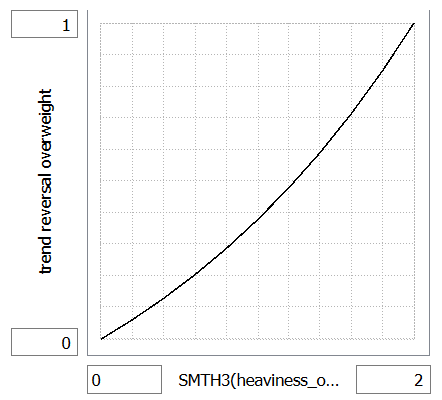 | Dimensionless | As the future is uncertain, the possibility to reverse the trend ‘overweight’, also to be adjusted by the policy lever ‘heaviness of trend reversal’. |
| 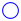 | "trend_reversal_on_-_heavy_overweight" | 0 | - | Dimensionless | **Policy lever** – the possibility to turn on or off the trend reversal. 1 is ‘trend conversion heavy overweight on’, whereas 0 is ‘trend conversion heavy overweight off’. |
| 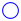 | "trend_reversal_on_-_obesity" | 0 | - | Dimensionless | **Policy lever** – the possibility to turn on or off the trend reversal. 1 is ‘trend conversion obesity on’, whereas 0 is ‘trend conversion obesity off’. |
| 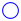 | "trend_reversal_on_-_overweight" | 0 | - | Dimensionless | **Policy lever** – the possibility to turn on or off the trend reversal. 1 is ‘trend conversion overweight on’, whereas 0 is ‘trend conversion overweight off’. |
|  | **Module – total cost** | | | | |
| 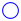 | Total_Cost_T2DM | Total_Fixed_Cost_per_T2DM_Patient  +Total_Variable_Cost_per_T2DM_Patient | - | Euros | The total costs, an accumulation of fixed and variable costs. |
| 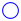 | Total_Costs_Elderly_Care_DM | ("Fraction_65+_Years_with_T2DM"  *Elderly_Care_DM)  *1.11125 | - | Euros/  Years | The total costs resulting from the care for elderly T2DM patients per T2DM patient.  *1.11125 is to make the variable for total DM pop (data missing) |
| 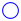 | "Total_Costs_for_Public_Authorities_per_T2DM_patient_(WIA-Benefits)" | (Total_Costs_WGA_Benefits_for_total_T2DM_Patients  +Total_Costs_IVA_Benefits_for_total_T2DM_Patient) | - | Euros/  persons/Years | The total costs for the Dutch public authorities resulting from providing WIA-benefits. |
| 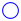 | Total_Costs_for_the_Employer | Total_Costs_of_Absenteeism_T2DM_Popn+  "Total_Costs_of_Labor_Productivity_Loss_T2DM_Patient_(WIA_employer)" | - | Euros/  Years | The total costs that are accounted to the employer due to T2DM per year |
| 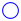 | Total_Costs_intervention_program_per_T2DM_patient | (Total_Costs_program_Execution_per_person*potential_T2DM_people_to_recruit_program)  *pricing_option | - | Euros/  Year | The total costs of lifestyle program per individual suffering from T2DM per year. |
| 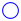 | Total_Costs_IVA_Benefits_for_total_T2DM_Patientt | ((Total_Costs_IVA_T2DM+Total_Costs_IVA_Execution_T2DM)/Total_Number_of_Persons_with_IVA)  *Number_of_T2DM_Patients_with_IVA | - | Euros/  Years | The total costs resulting from providing IVA-benefits per T2DM patient. |
| 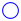 | Total_Costs_IVA_Execution_T2DM | 55538000 | - | Euros/  Years | Total execution costs of providing IVA-benefits for the T2DM population in 2013 (Rijksoverheid, n.d.). |
| 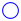 | Total_Costs_IVA_T2DM | 1119203000 | - | Euros/  Years | Total costs of providing IVA-benefits for the T2DM population in 2013 (Rijksoverheid, n.d.). |
| 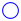 | Total_Costs_Management_Organizations_DM_Popn | 41400000 | - | Euros/  Years | Total costs for managing organizations concerned with DM (RIVM, 2013). |
| 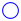 | Total_Costs_Management_Organizations_per_T2DM_patient | (Total_Costs_Management_Organizations_DM_Popn  *Fraction_T2DM_over_DM_Popn) | - | Euros/  persons/Years | Costs resulting from managing organizations concerned with T2DM. |
| 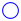 | Total_Costs_of_Absenteeism_T2DM_Popn | 186000000 | - | Euros/  Years | Total costs resulting from T2DM patients being absent as a consequence of T2DM  (KPMG, 2012, p. 16). |
| 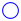 | "Total_Costs_of_Labor_Productivity_Loss_T2DM_Patient_(WIA_employer)" | 12000000 | - | Euros/  Years | Total costs resulting from labor productivity loss of the T2DM patient (KPMG, 2012, p. 16). |
| 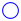 | Total_Costs_prevention_program_per_person | IF "Switch_program_(IFG/_IGT)"=1  THEN (Every_year_boost_prediabetes_program*Fraction_Dx_PreD_Popn_over_T2DM_Popn)  *pricing_option_prediabetes  ELSE 0 | - | Euros/  Years | The total costs resulting from executing LaM program as prevention program per diagnosed prediabetes (IFG) participant per year (TNO, 2016). That is, the total societal costs per T2DM patient times the number of IFG patients. It is assumed that the participation costs per T2DM patient is similar for the diagnosed prediabetes (IFG) patient. |
| 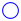 | Total_Costs_program_Execution_per_person | IF switch_program_T2DM=1  THEN (Boost_First_Year_Program_Costs_per_person+Every_Year_Boost_Program_Costs_per_person)  ELSE 0 | - | Euros/  Persons/Years | The total costs resulting from executing LaM as intervention program per T2DM participant per year (TNO, 2016). |
| 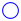 | Total_Costs_program_PreD_population | Total_Costs_prevention_program_per_person  *(Diagnosed_Prediabetes_Population*potential_people_to_recruit_program) | - | Euros/  Persons/Years | The total costs of LaM program per individual suffering from diagnosed prediabetes (IFG) per year (TNO, 2016). |
| 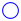 | Total_Costs_WGA_Benefits_for_total_T2DM_Patients | ((Total_Costs_WGA_T2DM+Total_Costs_WGA_Execution_T2DM)  /Total_Number_of_Persons_with_WGA)  *Ratio_of_T2DM_Patients_with_WGA | - | Euros/  Years | The total costs resulting from providing WGA-benefits per T2DM patient. |
| 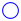 | Total_Costs_WGA_Execution_T2DM | 153230000 | - | Euros/  Years | Total execution costs of providing WGA-benefits for the T2DM population in 2013 (Rijksoverheid, n.d.). |
| 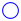 | Total_Costs_WGA_T2DM | 1946952000 | - | Euros/  Years | Total costs of providing WGA-benefits for the T2DM population in 2013 (Rijksoverheid, n.d.). |
| 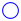 | Total_Elderly_Popn_2013 | 2121525+702820 | - | Persons | Total elderly population in The Netherlands in 2013. An individual is considered elderly from 65 years and older (CBS, 2017a). |
| 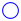 | Total_Fixed_Cost_per_T2DM_Patient | (T2DM_population_mutliplier)  *Total_Fixed_Cost_T2DM_Population | - | Euros/  Persons/Year | The total fixed cost per patient per year. |
|  | Total_Fixed_Cost_T2DM_Population | ("Total_Costs_for_Public_Authorities_per_T2DM_patient_(WIA-Benefits)"  +"Total_Medical_Costs_per_T2DM_Patient_(Coinsurance)"+ Total_Costs_Management_Organizations_per_T2DM_patient) | - | Euros/  Year | The total costs associated with the T2DM population per year. |
|  | "Total_Medical_Costs_per_T2DM_Patient_(Coinsurance)" | ((Total_Costs_Elderly_Care_DM+Hospitalization_DM+Primary_Care_DM+Ambulance_Care_DM+Tools_DM+Other_Health_Care_Providers_DM)*Fraction_T2DM_over_DM_Popn) | - | Euros/  persons/Year | The total medical costs resulting from individuals suffering from T2DM. |
|  | Ratio_of_T2DM_Patients_with_WGA | Total_Number_of_Persons_with_WGA*Fraction_T2DM_over_Total_Popn | - | Persons | The total number of individuals suffering from T2DM with WIA-benefits. Note: the number of individuals with WIA-benefits is determined in 2011 and the percentage individuals with T2DM is determined in 2000. |
|  | Total_Number_of_Persons_with_IVA | 47100 | - | Persons | Total number of individuals with IVA-benefits in the Netherlands in 2013. |
|  | Total_Number_of_Persons_with_WGA | 98150 | - | Persons | Total number of individuals with WGA-benefits in The Netherlands in 2013 (CBS, 2020). |
|  | Total_Obligatory_Continuation_of_Employees'_Salary_Payment_T2DM_Popn | Average_Obligatory_Continuation_of_Employee's_Salary_Payment_per_T2DM_Patient | - | Euros/  Years | The total costs resulting from paying the obligatory salary if an employee becomes disabled as a consequence of suffering from T2DM (CBP, 2012). |
|  | Total_Popn_2013 | 16779575 | - | Persons | Total population in the Netherlands in 2013 (CBS, 2020). |
|  | Total_Variable_Cost_per_T2DM_Patient | (Total_Obligatory_Continuation_of_Employees'_Salary_Payment_T2DM_Popn  *T2DM_Population  *Percentage_of_T2DM_'arbeidsongeschikt')  +(Total_Costs_for_the_Employer  +((Total_Costs_intervention_program_per_T2DM_patient*T2DM_Population)  +(Total_Costs_prevention_program_per_person  *T2DM_Population))) | - | Euros/  persons/Years | The total costs resulting from absenteeism of the T2DM patient and labor productivity loss of the T2DM patient per T2DM patient (NIVEL, 2012, p. 8). |
|  | Tools_DM | Devices_DM  +Medication_DM | - | Euros/  Years | Costs resulting from the need for tools and medication for the treatment and management of T2DM (RIVM, 2013). |
|  | Number_of_T2DM_Patients_with_IVA | Total_Number_of_Persons_with_IVA  *Fraction_T2DM_over_Total_Popn | - | Persons | The total number of individuals suffering from T2DM with IVA-benefits. Note: the number of individuals with IVA-benefits is determined in 2011 and the percentage individuals with T2DM is determined in 2000. |
|  | Other_Costs_DM | 295500000 | - | Euros/  Years | Total costs resulting from other primary care for DM patients per year (RIVM, 2013). |
|  | Other_Health_Care_Providers_DM | 3400000 | - | Euros/  Years | Total costs of other health care providers from having DM patients as clients per year (RIVM, 2013). |
|  | Percentage_of_T2DM_'arbeidsongeschikt' | 0.20 | - | Dimensionless | The percentage that is declared ‘unable to work’ because of their T2DM situation (2Diabeat, 2020) |
|  | Physiotherapy_DM | 900000 | - | Euros/  Years | Total costs resulting from physiotherapy for DM patients per year (RIVM, 2013). |
|  | Primary_Care_DM | GP_Care_DM  +Physiotherapy_DM  +Other_Costs_DM | - | Euros/  Years | Total costs resulting from primary care for T2DM patients (RIVM, 2013). 0.9: due to extracting the T2DM population of total costs |
|  | GP_Care_DM | 79100000 | - | Euros/  Years | Total costs resulting from DM patients needing general practitioners care per year (RIVM, 2013). |
|  | Hospitalization_DM | 180900000 | - | Euros/  Years | The total osts resulting from hospitalization due to DM per year (RIVM, 2013). |
|  | Medication_DM | 385600000 | - | Euros/  Years | Total costs resulting from the need for medication for the treatment and management of DM (RIVM, 2013). |
|  | Elderly_Care_DM | 456700000 | - | Euros/  Years | Costs resulting from the care for elderly DM patients (RIVM, 2013). |
|  | "Fraction_65+_Years_with_T2DM" | 0.109+0.111 | - | Dimensionless | The fraction elderly population suffering from T2DM in the Netherlands in 2013. An individual is considered elderly from 65 years and older (CBS, 2020). |
|  | Devices_DM | 244300000 | - | Euros/  Years | Total costs resulting from the need for tools for the treatment and management of DM (RIVM, 2013). |
|  | "'ziektewet-_uitkering'" | 0.7 | - | Percent | After 2 years of non-active service, oner receives a 70% pay-off every year of his/her last wage. |
|  | Ambulance_Care_DM | 900000 | - | Euros/  Years | Total costs resulting from ambulance care for DM patients per year (RIVM, 2013). |
|  | annual_salary | 36500 | - | Euros | The annual salary of the an average employee in the Netherlands (CBS, 2020). |
|  | annual_salary_ncrease | 0.02/12 | - | Percent/year | On average 2% raise over in current trends. This due to the exponential rise in wages in the last 10 years (2% on average) and the global competitiveness index  [Ontwikkeling cao-lonen (cbs.nl)](https://www.cbs.nl/nl-nl/visualisaties/dashboard-arbeidsmarkt/ontwikkeling-cao-lonen) ; [Global Competitiveness Index - TCdata360 (worldbank.org)](https://tcdata360.worldbank.org/indicators/gci) |
|  | Average_Obligatory_Continuation_of_Employee's_Salary_Payment_per_T2DM_Patient | (annual_salary+(annual_salary  *STEP(annual_salary_ncrease; 1)))  *"'ziektewet-_uitkering'" | - | Euros/  Persons/Years | Costs resulting from the obligatory payment of an employee’s salary when the employee becomes disabled as a consequence of T2DM per year. The average income for an average person per year  (CBP, 2012). |
|  | T2DM_population_mutliplier | GRAPH(T2DM_Population) |  | Dimensionless | On the basis of total fixed cost in 2013 (RIVM, 2013 – the current available data) one can derive a curve of cost per person via a multiplier. The more people have T2DM, the higher the cost.  NB: it might not be as linear, however this is an approximation as we are dealing with a large population. |
| **Module – prevention/ intervention programs** | | | | | |
|  | "Switch_program_(IFG/_IGT)" | IF TIME > 2020 THEN "program_on_(PreD)" ELSE 0 | - | Dimensionless | Switch program enables the effect of the prevention program. The switch can only take on the values 0 and 1: no implementation of a certain program and implementation of a certain program, respectively. |
|  | switch_program_T2DM | IF TIME > 2020 THEN "Program_on_(T2DM)"ELSE 0 | - | Dimensionless | Switch program enables the effect of the intervention program. The -switch can only take on the values -0 and 1: no implementation of a certain program and implementation of a certain program, respectively. |
|  | "program_on_(PreD)" | 1 | - | Dimensionless | **Policy lever –** the activation of any of the prevention programs. 1 is ‘prevention program on’, whereas 2 is ‘prevention program off’. |
|  | "Program_on_(T2DM)" | 1 | - | Dimensionless | **Policy lever –** the activation of any of the intervention programs. 1 is ‘intervention program on’, whereas 2 is ‘intervention program off’. |
|  | Boost_First_Year_Program_Costs_per_person | PULSE(1585; 2020; 10000) | - | Euros  /Years | Boost first year to be able to execute LaM program (TNO, 2016). |
|  | "Maximum_Patients_able_to_treat_(IFG)" | Diagnosed_Prediabetes_Population  *"Switch_program_(IFG/_IGT)" | - | Persons/Years | The maximum diagnosed prediabetes (IFG) patients that can be treated per year. |
|  | "Maximum_Patients_able_to_treat_(T2DM)" | (T2DM_Population/"Treatment_Time_(T2DM)")  *switch_program_T2DM | - | Persons/Years | The maximum T2DM patients that can be treated per year. |
|  | potential_people_to_recruit_program | 0.9 | - | Dimensionless | **Policy lever -** Percentage to adjust; the amount of people that can be recruited for a program |
|  | "Potential_Recruitment_Rate_(IFG)" | "Maximum_Patients_able_to_treat_(IFG)"  *potential_people_to_recruit_program | - | Persons/Years | The rate which shows the potential of diagnosed prediabetes (IFG) patients that can be recruited per year. |
|  | "Potential_Recruitment_Rate_(T2DM)" | "Maximum_Patients_able_to_treat_(T2DM)"  *potential_T2DM_people_to_recruit_program | - | Persons/Years | The rate which shows the potential of T2DM patients that can be recruited per year. |
|  | potential_T2DM_people_to_recruit_program | 1 | - | Dimensionless | **Policy lever –** The potential amount of people that can be -recruited for a program. This is a weigh-off. The more people that can be recruited, the more the cost most likely. The policy is a slider from 0-1 where 0 is no people from the T2DM stock, and 1 is all people from the T2DM stock. |
|  | pricing_option | 1 | - | Dimensionless | **Policy lever –** The pricing of the intervention programs can be adjusted. The standard is the LaM program (TNO, 2016) . The slider can be adjusted from 0 to infinity; whereas 1 is the standard amount and for example 3 is three times the amount of the LaM program. |
|  | pricing_option_prediabetes | 1 | - | Dimensionless | **Policy lever –** The pricing of the prevention programs can be adjusted. The standard is the LaM program (TNO, 2016) . The slider can be adjusted from 0 to infinity; whereas 1 is the standard amount and for example 3 is three times the amount of the LaM program (TNO, 2016). |
|  | Every_Year_Boost_Program_Costs_per_person | STEP(400; 1) | - | Euros  /persons/Years | Every year boost to be able to execute LaM as intervention program (TNO, 2016). This is chosen to be a standard cost program. |
|  | "program_on_(PreD)" | 1 | - | Dimensionless | **Policy lever –** the activation of any of the prevention programs. 1 is ‘prevention program on’, whereas 2 is ‘prevention program off’. |
|  | "Program_on_(T2DM)" | 1 | - | Dimensionless | **Policy lever –** the activation of any of the intervention programs. 1 is ‘intervention program on’, -whereas 2 is ‘intervention program off’. |
|  | "program_Success_Rate_prediabetes" | 0,8 | - | Dimensionless | Policy lever- The success rate determines the number of diagnosed prediabetes (IFG) patients that are successfully reversed to healthy individuals again when participating in the prevention program LaM per year (TNO, 2016, p.10). It is assumed to be similar to the T2DM population. 0 is completely no success (all patients remain in the prediabetes stock, 1 is complete successs (all diagnosed prediabetes patients reverse to the normoglycemic stock). |
|  | "program_Success_Rate_(T2DM)" | 1 | - | Dimensionless | **Policy lever -** The success rate determines the number of T2DM patients that are successfully reversed to healthy individuals again when participating in the intervention program per year. 0 is ‘no success at all’ whereas 1 is ‘all patients successful in flowing to no medicine necessary stock’. |
|  | ratio_extra_recovery_rate | SMTH3(Extra_recovery_rate_Intervention_Program_T2DM/T2DM_Population; 10 ) | - | Dimensionless | The ratio in which the intervention program is implemented – from 0 to 10 year the program is in full effect. |

**Modules: The patient Journey**

**Module: Intervention and prevention programs**

**Module: CostModule: overweight trends**
